# M2 Macrophage‐Derived sEV Regulate Pro‐Inflammatory CCR2^+^ Macrophage Subpopulations to Favor Post‐AMI Cardiac Repair

**DOI:** 10.1002/advs.202202964

**Published:** 2023-03-22

**Authors:** Lan Li, Jiasong Cao, Sheng Li, Tianyi Cui, Jingyu Ni, Han Zhang, Yan Zhu, Jingyuan Mao, Xiumei Gao, Adam C. Midgley, Meifeng Zhu, Guanwei Fan

**Affiliations:** ^1^ State Key Laboratory of Modern Chinese Medicine Key Laboratory of Pharmacology of Traditional Chinese Medical Formulae for the Ministry of Education Tianjin University of Traditional Chinese Medicine Tianjin 301617 China; ^2^ National Clinical Research Center for Chinese Medicine Acupuncture and Moxibustion State Key Laboratory of Component‐based Chinese Medicine First Teaching Hospital of Tianjin University of Traditional Chinese Medicine Tianjin 300193 China; ^3^ Tianjin Key Laboratory of Human Development and Reproductive Regulation Tianjin Central Hospital of Gynecology Obstetrics Tianjin 300052 China; ^4^ Key Laboratory of Bioactive Materials for the Ministry of Education College of Life Sciences Nankai University Tianjin 300071 China

**Keywords:** CC chemokine receptor 2, extracellular vesicles, ischemia‐reperfusion injury, macrophage metabolic reprogramming, macrophages

## Abstract

Tissue‐resident cardiac macrophage subsets mediate cardiac tissue inflammation and repair after acute myocardial infarction (AMI). CC chemokine receptor 2 (CCR2)‐expressing macrophages have phenotypical similarities to M1‐polarized macrophages, are pro‐inflammatory, and recruit CCR2^+^ circulating monocytes to infarcted myocardium. Small extracellular vesicles (sEV) from CCR2^–^ macrophages, which phenotypically resemble M2‐polarized macrophages, promote anti‐inflammatory activity and cardiac repair. Here, the authors harvested M2 macrophage‐derived sEV (M2_EV_) from M2‐polarized bone‐marrow‐derived macrophages for intramyocardial injection and recapitulation of sEV‐mediated anti‐inflammatory activity in ischemic‐reperfusion (I/R) injured hearts. Rats and pigs received sham surgery; I/R without treatment; or I/R with autologous M2_EV_ treatment. M2_EV_ rescued cardiac function and attenuated injury markers, infarct size, and scar size. M2_EV_ inhibited CCR2^+^ macrophage numbers, reduced monocyte‐derived CCR2^+^ macrophage recruitment to infarct sites, induced M1‐to‐M2 macrophage switching and promoted neovascularization. Analysis of M2_EV_ microRNA content revealed abundant miR‐181b‐5p, which regulated macrophage glucose uptake, glycolysis, and mitigated mitochondrial reactive oxygen species generation. Functional blockade of miR‐181b‐5p is detrimental to beneficial M2_EV_ actions and resulted in failure to inhibit CCR2^+^ macrophage numbers and infarct size. Taken together, this investigation showed that M2_EV_ rescued myocardial function, improved myocardial repair, and regulated CCR2^+^ macrophages via miR‐181b‐5p‐dependent mechanisms, indicating an option for cell‐free therapy for AMI.

## Graphical abstract

M2 macrophage‐derived small extracellular vesicles (M2_EV_) regulated metabolic reprogramming of CC chemokine receptor 2 (CCR2)^+^ macrophages by delivery of miR‐181b‐5p, which in turn prevented the CCR2^+^ > CCR2^−^ macrophage imbalance observed after ischemic‐reperfusion (I/R) injury induced myocardial infarction. This results in the inhibition of the recruitment of CCR2^+^ monocytes and monocyte‐derived macrophages to the myocardium, thereby promoting cardiac tissue repair.

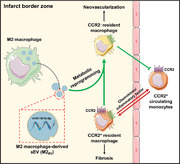

## 1 Introduction

Acute myocardial infarction (AMI) is the leading cause of mortality worldwide.^[^ ^1^ ^]^ Percutaneous coronary intervention (PCI) is currently the most effective strategy to achieve timely revascularization and limit AMI‐related issues.^[^ ^2^ ^]^ However, incomplete reperfusion and large or multiple infarction sites lead to left ventricular (LV) remodeling, which promotes progressive heart failure, manifesting clinically as ischemia‐reperfusion (I/R) injury.^[^ ^3^ ^]^ In 2007, a randomized controlled trial published in the “New England Journal of Medicine” reported that PCI did not reduce the risk of death, myocardial infarction, or other major cardiovascular events.^[^ ^4^ ^]^ LV remodeling after reperfusion is closely tied to macrophage‐mediated inflammatory responses. Macrophages determine the development direction of aggravation or repair of heart injury after AMI according to their source, location, quantity, and phenotype.^[^ ^5^ ^]^ Post‐I/R injury, an early inflammatory phase exacerbated by neutrophils and inflammatory monocytes, provokes the chronic activation of pro‐inflammatory monocyte‐derived M1 macrophages, which in turn can result in tissue destruction.^[^ ^5^, ^6^ ^]^ Under physiological resolution of tissue inflammation, M1 macrophages implement a transition to reparative M2 macrophage phenotypes, which promote tissue regeneration by cell‐cell orchestration of endothelial cells, fibroblasts, parenchymal, and local progenitor cells.^[^ ^7^ ^]^ The combined M1 macrophage abundance and non‐selective depletion of M2 macrophages ultimately limit cardiac repair and function through excessive inflammation‐induced tissue damage and scar formation.^[^ ^8^ ^]^ However, M1/M2 classification alone is not sufficient to clarify the diversity of macrophages after cardiac I/R injury.

Previously, functionally distinct subsets of macrophages that reside within the myocardium under steady‐state conditions with differential expression of CC chemokine receptor 2 (CCR2), were identified to have distinct phenotypic characteristics and roles in myocardial inflammation.^[^ ^9^ ^]^ Tissue‐resident cardiac macrophages can be divided into CCR2^−^ and CCR2^+^ subsets derived from embryonic and adult hematopoietic lineages, respectively.^[^ ^10^ ^]^ Tissue‐resident cardiac CCR2^+^ macrophages are initially derived from circulating hematopoietic progenitors and monocytes, whereas CCR2^−^ macrophages seed the myocardium during the first few weeks of embryonic development.^[^ ^11^ ^]^ In response to I/R injury, resident CCR2^+^ macrophages recruit circulating CCR2^+^ monocytes to the infarcted myocardium through myeloid differentiation primary response 88 (MYD88)‐dependent pathways and they upregulate the secretion of proinflammatory cytokines and chemokines, such as monocyte chemoattractant protein‐1 (MCP‐1; CCL2).^[^ ^12^ ^]^ CCR2^+^ monocytes exhibit pro‐inflammatory activity and differentiate into monocyte‐derived CCR2^+^ macrophages with similar phenotypical characteristics to classically described M1 macrophages; and have been implicated in the promotion of pathogenic adverse cardiac remodeling.^[^ ^13^ ^]^ In contrast, the CCR2^−^ macrophage population is maintained in the myocardium independent of peripheral blood monocyte input and under steady‐state conditions. CCR2^−^ macrophages were shown to promote coronary development and cardiac regeneration.^[^ ^12^, ^14^ ^]^ Although extensive research has provided evidence to support distinct roles relating to CCR2^+^ and CCR2^−^ resident macrophage imbalance during cardiac injury, the regulatory mechanisms, and interactions between different subtypes of circulation‐derived macrophages and resident macrophages remain unclear.

Small extracellular vesicles (sEV) are a class of cell‐derived vesicles that are known to convey intercellular communications and facilitate the exchange of cellular substances and information. Among the proteins and nucleic acids transported by sEV, microRNA (miRNA) content was shown to feature prominently in the modulation of intercellular communications by influencing epigenetic regulation in receiving cells.^[^ ^15^ ^]^ Therapies based on sEV have received increasing attention as cell‐free approaches to regenerative medicine, particularly in the context of cardioprotective potential and the rescue of cardiac function in animal models of AMI.^[^ ^16^ ^]^ The release of sEV by reparative macrophage phenotypes (M2 macrophages) has been suggested to be a key mechanism by which resident macrophages are modulated to exert anti‐inflammatory effects in the mediation of cardiac repair.^[^ ^17^ ^]^ Thus, isolation, content characterization, and application of autologous M2 macrophages may offer an additional cell source for therapeutic sEV harvest and to realize immunomodulation beneficial to the AMI heart; whilst demonstrating cell‐cell communication mechanisms that govern distinct macrophage subpopulation actions during cardiac damage versus cardiac repair.

In this investigation, we assessed the plasticity and pro‐reparative effects of M2‐derived sEV (M2_EV_) in rat and porcine models of myocardial I/R injury. We observed that M2_EV_ prevented CCR2^+^ > CCR2^−^ macrophage imbalance and inhibited the recruitment of CCR2^+^ monocytes and monocyte‐derived macrophages to the myocardium following I/R injury, which promoted cardiac repair by enhancement of revascularization. We subsequently explored the mechanisms that governed macrophage phenotypic changes related to cardioprotective effects through the assessment of M2_EV_ miRNA content and associated cellular functions. The reparative mechanisms of M2_EV_ shown here help elucidate intercellular communications and regulatory signals between different macrophage subpopulations and suggested that M2_EV_ and CCR2^+^ cells represent promising targets to suppress inflammation and adverse remodeling in patients with myocardial infarction or heart failure.

## 2 Results

### 2.1 Characterization of M2 Macrophage‐Derived sEV

Rat bone marrow‐derived macrophages (BMDM) were treated with interleukin (IL)‐4/IL‐13 or lipopolysaccharide (LPS)/interferon (IFN)‐*γ* to direct them to M2 or M1 macrophages, respectively^[^ ^18^ ^]^ (Figure S1A, Supporting Information). Small extracellular vesicles (sEV) were isolated from the conditioned medium of rat M2 macrophage cultures by ultracentrifugation. The morphology and size of the M2 macrophage‐derived sEV (M2_EV_) were assessed by transmission electron microscopy (TEM) and nano‐flow cytometry (NanoFCM). The isolated M2_EV_ and M1_EV_ exhibited typical membrane structures with approximate dry‐state diameters < 150 nm, as observed by TEM (Figure S1B, Supporting Information). The NanoFCM identified and measured the diameter size of M2_EV_ and M1_EV_, which averaged 79.8 ± 15.5 and 77.8 ± 21.3 nm, respectively (Figure S1D,E, Supporting Information). Expression of EV marker proteins (Alix, CD9, CD63) and absence of the endoplasmic reticulum protein calnexin were confirmed by Western blot (Figure S1C, Supporting Information), indicative of successful sEV fraction isolation. The lipophilic membrane dye, PKH26, was used to label M2_EV_ prior to their incubation with RAW264.7 macrophages. RAW264.7 macrophages demonstrated the ability of M2_EV_ uptake (Figure S1F, Supporting Information). We next sought to evaluate the time of residence in the heart, following intramyocardial injection into rats. The results demonstrated that M2_EV_ presence could still be detected after 72 h and a gradual linear decrease in radiant efficiency was observed across the 72‐h time course (Figure S1G, Supporting Information). These data suggested that M2_EV_ were either being taken up by tissues in vivo or remained in circulation for at least 72 h. Additionally, porcine‐ and mouse‐sourced M2_EV_ were assessed using TEM and NanoFCM to determine whether there was species variance in sEV size. The mean diameter of porcine‐sourced M2_EV_ was 81.8 ± 21.3 nm (Figure S1H,I, Supporting Information), and the mean diameter of mouse‐sourced M2_EV_ was 74.2 ± 18.4 nm (Figure S1H,J, Supporting Information), which indicated that macrophage‐derived sEV from different animal models exhibited no statistically significant differences in mean diameter size. Taken together, our characterization data indicated that M2_EV_ was successfully obtained and characterized for subsequent use in functional verification experiments.

### 2.2 M2_EV_ Treatment Rescues Myocardial Function in Rat Models of I/R Injury

**Figure 1:**
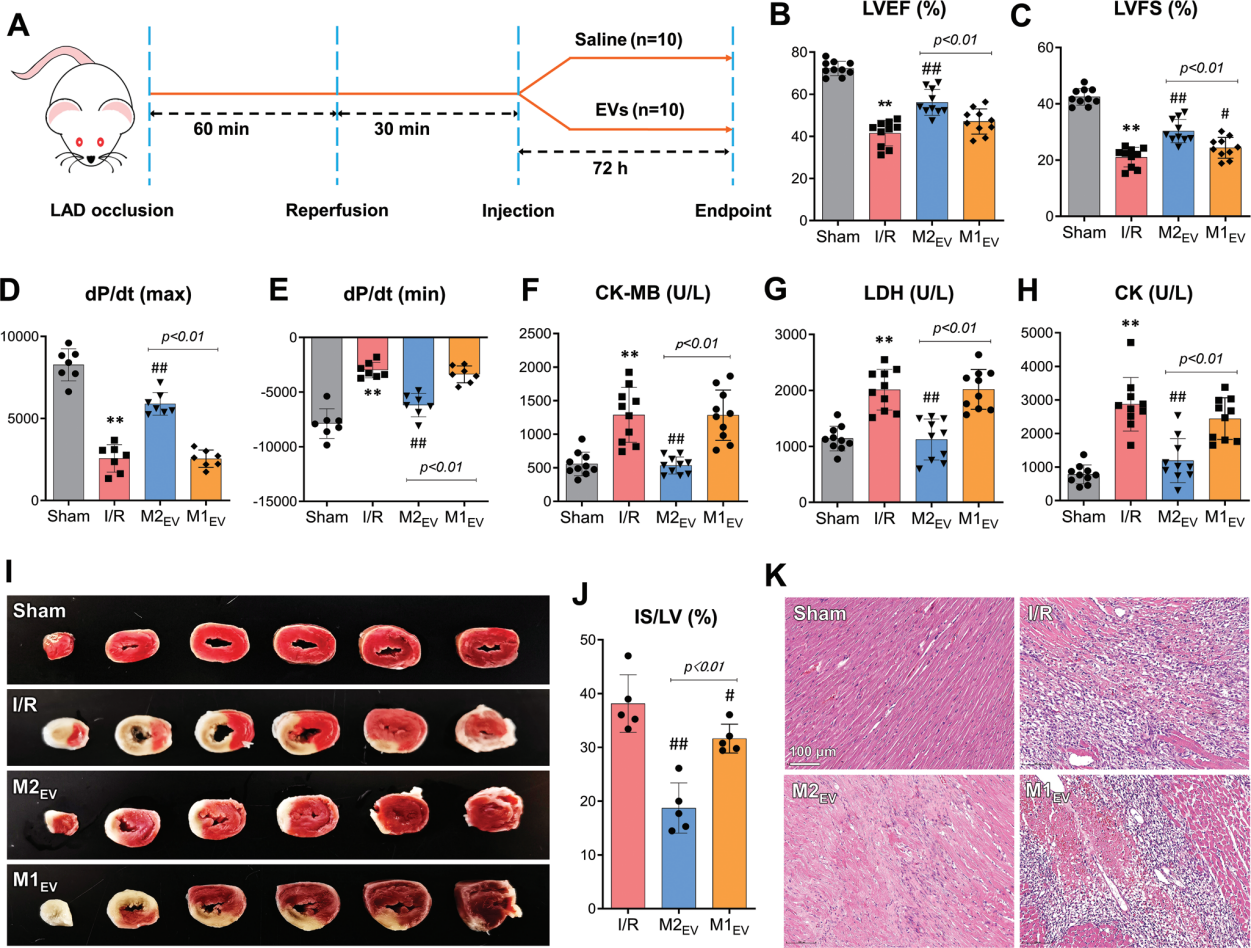
Protective activity of intramyocardially administered M2_EV_ in I/R rat hearts. A) Schematic overview of the experimental procedure. Quantification of B) LVEF % and C) LVFS % 3 days after I/R, data are presented as mean ± SD of *n* = 10 rats. D,E) Quantification of maximal and minimal LV pressure derivative (dP/dt), data are presented as mean ± SD of *n* = 7 rats. F–H) Blood serum analyses of LDH, CK, and CK‐MB enzyme levels, respectively, data are presented as mean ± SD of *n* = 10 rats. I,J) Representative TTC‐stained heart images and quantified IS % (rat hearts were cut into six transverse slices (from apex to basal edge of infarction) 3 days after I/R, data are presented as mean ± SD of *n* = 5 rats. K) Representative H&E staining of heart transverse sections (scale bar = 100 µm) 3 days after I/R. Statistical significance is indicated as **p < 0.01 compared with the sham group and ^##^ *p* < 0.01/^#^ *p* < 0.05 compared with the I/R group.

The therapeutic efficacy of M2_EV_ was assessed in rat models of myocardial ischemia‐reperfusion (I/R) injury (**Figure** **1**A). M2_EV_ were intramyocardially administered to rats 30 min after the time of reperfusion. EV‐free saline and M1_EV_ served as the controls. LV contractility and function were improved in I/R rats receiving M2_EV_ treatment, as evidenced by significantly improved ejection fraction (LVEF; Figure 1B) and fractional shortening (LVFS; Figure 1C) measurements, 72 h after injection. In addition, we also observed that M1_EV_ significantly improved LVFS. Maximal and minimal left ventricular pressure derivative (dP/dt) was measured by invasive hemodynamic assessment of pressure‐volume (PV) loops. M2_EV_ treatment significantly improved dP/dt max (Figure 1D) and reduced dP/dt min (Figure 1E), compared to the I/R and M1_EV_ groups. The serum levels of lactate dehydrogenase (LDH), creatine kinase (CK), and CK MB fraction (CK‐MB) enzymes were evaluated, which are indicative of enzyme release from the cytosol of cardiomyocytes into the blood following myocardial injury.^[^ ^19^ ^]^ The presence of LDH (Figure 1E), CK (Figure 1F), and CK‐MB (Figure 1G) were all significantly decreased in the blood serum of I/R rats treated with M2_EV_, compared to I/R rats without treatment or M1_EV_ treatment. Excised hearts were assessed for infarct size (LV/IS) by 2,3,5‐triphenyltetrazolium chloride (TTC) staining (Figure 1I) and quantified relative to the area occupied by infarcted tissue (Figure 1J). I/R rats that received M2_EV_ and M1 _EV_ treatments had significantly decreased LV/IS (≈38.1% in I/R hearts, 18.7% in M2_EV_‐treated I/R hearts, and 31.6% in M1_EV‐_treated I/R hearts). Additional analysis by hematoxylin and eosin (H&E) staining showed a large influx of inflammatory cells into the IS in I/R and M1_EV_‐treated rat myocardial tissue, whereas M2_EV_ inhibited the infiltration of these monocyte cells into the IS (Figure 1K). Taken together, these data indicated that M2_EV_ exerted a protective activity against I/R heart damage.

### 2.3 M2_EV_ Treatment Improves Myocardial Function in Porcine Models of I/R Injury

**Figure 2:**
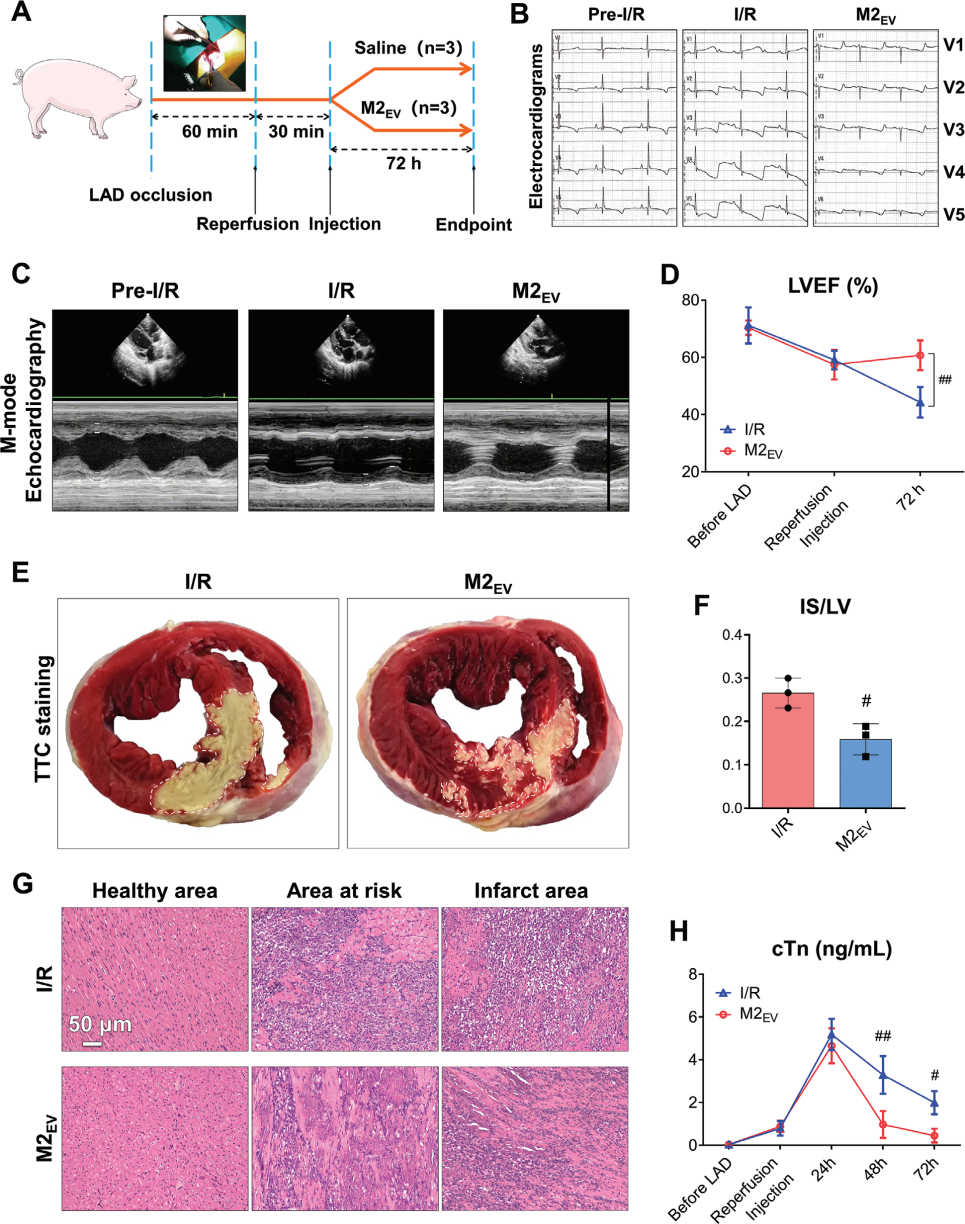
M2EV demonstrates a cardioprotective effect against I/R injury in porcine models. A) Schematic of the experimental procedure. B) ECG readings pre‐I/R and at experimental endpoints. C) Representative M‐mode echocardiography images. D) Quantification of LVEF %. E) TTC staining of transverse heart sections to visualize the IS area in pig hearts. F) IS quantification. G) Representative H&E staining of healthy tissue, the area at the risk border zone, and the IS area (scale bar = 50 µm). H) Levels of cTn (ng mL^−1^) measured in pig plasma at the indicated stages of the experimental procedure. All data are presented as mean ± SD of *n* = 3 pigs. Statistical significance is shown as ^##^ *p* < 0.01 and ^#^ *p* < 0.05 compared with the I/R group.

We next sought to determine whether M2_EV_ treatments could demonstrate a protective effect against I/R injury in larger animal models. Therefore, myocardial I/R injury was performed in porcine models utilizing an equivalent surgical procedure to the previously used rat models (**Figure** **2**A). Heart function was evaluated by electrocardiogram (ECG) analysis, 72 h after intramyocardially administered saline control or M2_EV_ treatments (Figure 2B). At 72‐h post‐reperfusion, pigs exhibited an ECG reading characteristic of AMI, which was improved following M2_EV_ treatment. M‐mode echocardiography (Figure 2C) enabled the quantification of LVEF (Figure 2D), which showed that I/R hearts that received M2_EV_ treatment had significantly improved function compared to I/R hearts that received saline. TTC staining (Figure 2E) demonstrated a similar trend to the reductions of IS area, as observed in rat models. M2_EV_ treatments significantly prevented the progression of myocardial infarction and IS area after I/R (Figure 2F). H&E staining of heart cross‐sections showed that M2_EV_ reduced the overall immune cell infiltration from the IS to the area at risk border zone (Figure 2G). An increased level of plasma cardiac troponin (cTn) is used as a marker of established myocardial infarction. In the I/R pig models, cTn concentration peaked at 24 h and was maintained at a high level for the subsequent 48 h. When M2_EV_ treatment was administered to I/R hearts, the plasma cTn levels also peaked at 24 h and showed no significant differences. However, cTn was significantly reduced at 48 and 72 h in I/R hearts that received M2_EV_, compared to I/R hearts that received saline injections (Figure 2H). Our results suggested that M2_EV_ treatments maintained a cardioprotective effect against I/R injury in the large pig models of myocardial infarction.

### 2.4 M2_EV_ Repress CCR2^+^ Macrophage Numbers in Favor of Reparative Phenotype Macrophages

**Figure 3:**
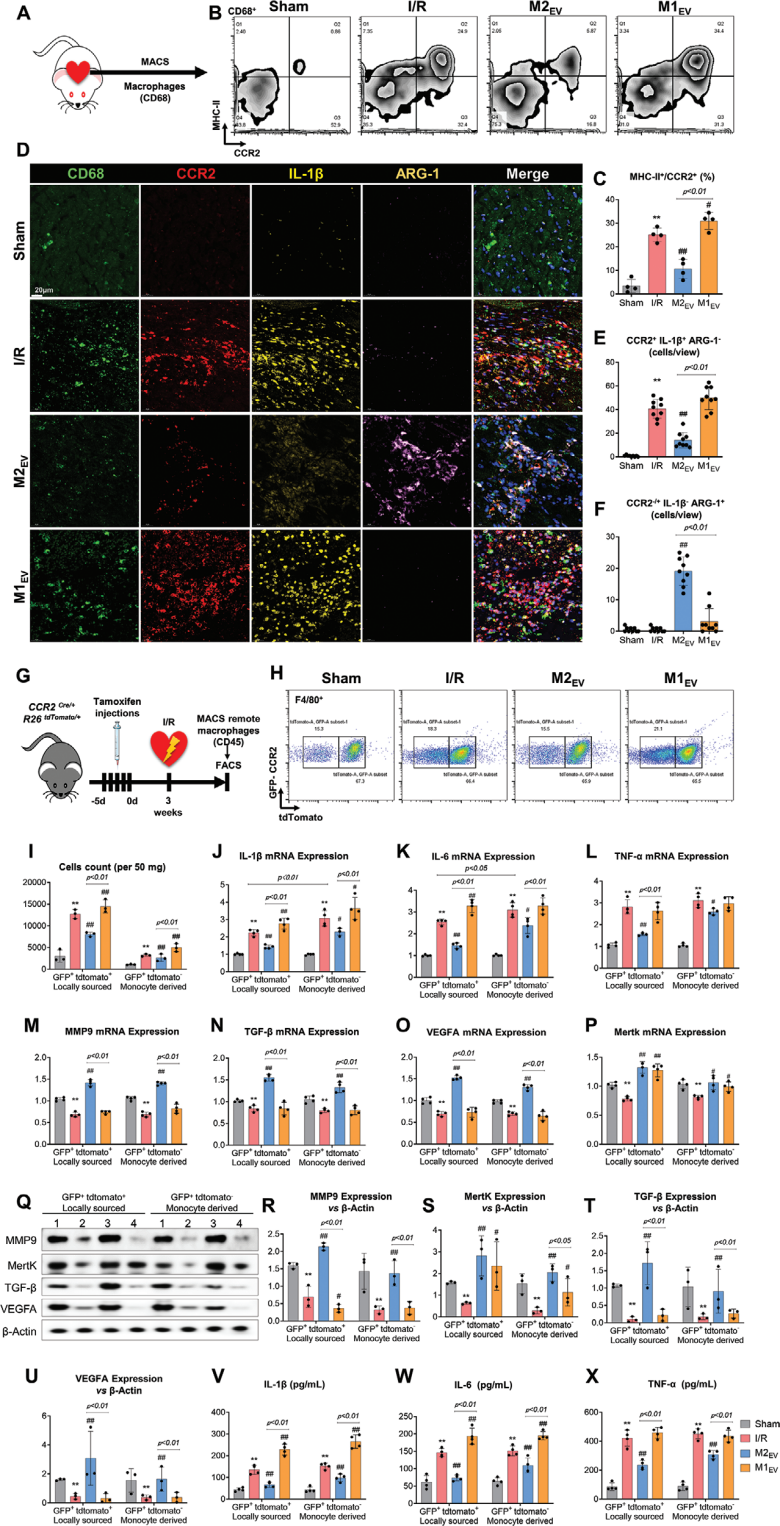
M2_EV_ inhibits CCR2^+^ macrophage presence in I/R hearts. A) Schematic of the experimental approach. B,C) Flow cytometry analysis, and quantification of MHC‐II^Hi^/CCR2^+^ cells, respectively, *n* = 4. D) Representative immunostaining of CD68, CCR2, ARG‐1, and IL‐1*β*. E,F) Quantitative analysis of CCR2^+^ IL‐1*β* ^+^ ARG‐1^−^, and CCR2^−/+^ IL‐1*β* ^−^ ARG‐1^+^ macrophages from stained tissue sections in D (*n* = 3 rats in each group, three microscopic fields of view for each sample, scale bar = 20 µm). G) Experimental design for tamoxifen‐treated CCR2^CreER/+^ R26^tdtomato/+^ mice to deplete CCR2^+^ circulating monocytes. H,I) Quantification of resident and monocyte‐derived cardiac CCR2^+^ macrophages, hearts from 6–8 mice were mixed as one sample, *n* = 3. J–P) Phenotyping of tissue‐resident and monocyte‐derived cardiac CCR2^+^ macrophages using RT‐PCR to assess gene expression from fate mapping experiments outlined in G, *n* = 4. Q–U) The protein levels of matrix metalloproteinase 9 (MMP9), transforming growth factor (TGF)‐*β*, vascular endothelial growth factor A (VEGFA), and Mertk in tissue‐resident and monocyte‐derived cardiac CCR2^+^ macrophages using Western blot, *n* = 3. V–X) The protein levels of IL‐1*β*, IL‐6, and tumor necrosis factor (TNF)‐*α* in tissue‐resident and monocyte‐derived cardiac CCR2^+^ macrophages using ELISA, *n* = 4. All data are presented as the mean ± SD. Statistical significance is shown as **p < 0.01 compared with the sham group and ^##^ *p* < 0.01 compared with the I/R group.

Considering the observations made in H&E sections from both animal models, we assessed changes in resident CCR2^+^ macrophage populations, the pro‐inflammatory macrophages that express CCR2 receptors. Our previous experiments demonstrated that although CCR2^+^ macrophages are present in the healthy myocardium, their increased numbers have been described to be closely related to cardiac inflammation and repair processes.^[^ ^20^ ^]^ Following myocardial injury, the macrophage population occupied by resident cardiac major histocompatibility complex (MHC)‐II^Hi^/CCR2^−^ macrophages becomes outnumbered by CCR2^+^ macrophages (MHC‐II^Hi^/CCR2^+^), leading to the subsequent increase in the production of pro‐inflammatory IL‐1*β*; which is widely associated with the promotion of cardiovascular diseases.^[^ ^12b^ ^]^ CD68^+^ macrophages were purified from homogenized risk border zone tissue by magnetic‐activated cell sorting (MACS) of single‐cell suspensions (**Figure** **3**A). The expression levels of CCR2 and MHC‐II were then assessed by flow cytometry. Analysis showed that M2_EV_ treatments significantly reduced the counts of MHC‐II^Hi^/CCR2^+^ macrophage (Figure 3B,C, and Figure S4A, Supporting Information). The MHC‐II^Hi^/CCR2^+^ macrophage fraction was markedly increased in I/R hearts, and the hearts receiving M2_EV_ treatments attenuated the increase in MHC‐II^Hi^/CCR2^+^ macrophage proportions. In addition, M1_EV_ treatments significantly induced the counts of MHC‐II^Hi^/CCR2^+^ macrophage compared to I/R (Sham 3.2 ± 2.8%; I/R 25.1 ± 2.8%; M2_EV_ 10.6 ± 4.0%; M1_EV_ 31.0 ± 3.6%) (Figure 3C). PKH26‐labeled M2_EV_ were intramyocardially injected, and following excision and tissue sectioning, sections were co‐stained with CCR2. The results showed that M2_EV_ was uptake by CCR2^+^ macrophage (Figure S2A, Supporting Information). Co‐immunostaining for CCR2 and CD68 in porcine myocardial tissue sections reciprocated these findings. CCR2^+^ macrophages were reduced in area at risk and infarct areas of porcine cardiac tissue sections, and CD68^+^ macrophage recruitment was significantly attenuated in the area at risk and infarct area, following M2_EV_ treatment (Figure S2B–D, Supporting Information). Constructive remodeling macrophage phenotypes (CD68^+^ CCR2^+/−^ arginase (ARG)‐1^+^; resemblance to M2 phenotypes) and pro‐inflammatory macrophage phenotypes (CD68^+^ CCR2^+^ IL1‐*β* ^+^; resemblance to M1 phenotypes) were identified by immunostaining (Figure 3D). Quantification results revealed that the numbers of CCR2^+^ IL1‐*β* ^+^ macrophages decreased within the area at the risk border zone, whereas the numbers of CCR2^+/−^ ARG‐1^+^ macrophages increased in the same area, after M2_EV_ treatment (Figure 3E,F). In addition, M1_EV_ did not cause significant changes in the number of CCR2^+^ IL1‐*β* ^+^ macrophages and CCR2^+/−^ ARG‐1^+^ macrophages but demonstrated a trend toward the upregulation of these two macrophage types (Figure 3E,F). In vitro experiments demonstrated that incubation of M2_EV_ with BMDM inhibited LPS+IFN*γ* induced NF*κ*B nuclear translocation (Figure S3A, Supporting Information). Furthermore, BMDM were stained for the M1 and M2 macrophage markers, iNOS and ARG1, respectively. Incubation with M2_EV_ inhibited M1 macrophage activation by LPS+IFN*γ* stimulation, and markedly induced M2 macrophage polarization (Figure S3B, Supporting Information). Assessment of mRNA expression confirmed that M2_EV_ attenuated LPS+IFN*γ* induced expression of pro‐inflammatory factors (*Il1b*, *Il6*, *Tnfa*) and the M1 marker (*Nos2*) (Figure S3C, Supporting Information).

To further determine whether the accumulation of pro‐inflammatory CCR2^+^ macrophages was dependent on the recruitment of circulating monocytes or the proliferation of tissue‐resident cardiac monocytes, we performed fate mapping using CCR2^CreER/+^ R26^tdtomato/+^ mouse models (Figure 3G). In these mice, all CCR2 receptor‐expressing cells, including circulating monocytes and tissue‐resident cardiac macrophages, express green fluorescent proteins (GFPs). Following tamoxifen administration, all CCR2^+^ cells also express a red fluorescent protein (tdTomato). Therefore, all blood CCR2^+^ monocytes and tissue‐resident CCR2^+^ macrophages co‐expressed green and red fluorescence. After three weeks, circulating monocyte populations were replaced by hematopoietic progenitor cell‐derived CCR2^−^ monocytes. Thus, these circulating monocytes no longer expressed tdTomato but continued to express GFP (Figure S4B, Supporting Information), whereas macrophages derived from proliferating resident CCR2^+^ macrophages continue to co‐express tdTomato and GFP. Following I/R injury, CD45^+^ cells were purified from single cell suspensions of CCR2^CreER/+^ R26^tdTomato/+^ infarction border zone tissue by MACS. Blood monocyte‐derived CCR2^+^ macrophages (GFP^+^ tdTomato^−^) and tissue‐resident CCR2^+^ macrophages (GFP^+^ tdTomato^+^) were quantified and subsequently sorted by fluorescence‐activated cell sorting (FACS) (Figure 3H,I, and Figure S4B, Supporting Information). Due to the relative scarcity of CCR2^+^ macrophages in mice hearts, the hearts from 6–8 mice were combined for analysis. The results showed that monocyte‐derived and locally proliferating CCR2^+^ macrophages both increased significantly after I/R injury, that M2_EV_ inhibited CCR2^+^ macrophages arising from both sources, and that M1_EV_ further increased the number of differentially sourced CCR2^+^ macrophages compared with I/R group (Figure 3H,I)_._

We next sought to determine whether M2_EV_ affected differentially sourced CCR2^+^ macrophages by using reverse transcription quantitative real‐time PCR (RT‐qPCR) to assess macrophage gene expression. Monocyte‐derived macrophages tended to exhibit greater inflammation‐associated gene expression than tissue‐resident macrophages after I/R injury, whereas the expression of inflammation‐associated genes in macrophages from both sources decreased significantly after M2_EV_ treatment. In addition, M1_EV_ further increased inflammation‐associated gene expression in both sources of CCR2^+^ macrophages (Figure 3J–L). The results suggested that regardless of the CCR2^+^ macrophage source, post‐I/R injury delivery of M2_EV_ suppressed the polarization of CCR2^+^ macrophage to phenotypes akin to M2 macrophages. In contrast, M1_EV_ promoted the polarization of CCR2^+^ macrophages to M1‐like macrophages. The expression of tissue repair/remodeling related genes (MMP9, TGF‐*β*, VEGFA) in monocyte‐derived and tissue‐resident macrophages were decreased following I/R injury, and M2_EV_ treatment ameliorated the expression of these genes (Figure 3M–O). Reparative phenotypes (generalized as M2 macrophages) have been ascribed to tissue repair/remodeling after 7 days post‐injury.^[^ ^6^ ^]^ Our results suggested that M2_EV_ treatments had the capacity to induce the earlier arrival of reparative phenotypes. Interestingly, Both M2_EV_ and M1_EV_ induced expression of the phagocytosis‐related gene, *Mertk*, in monocyte‐derived CCR2^+^ macrophages and tissue‐resident CCR2^+^ macrophages (Figure 3P). These results revealed a potential explanation as to why M1_EV_ was effective in diminishing the infarct size after I/R (Figure 1I,J). M1_EV_ contributes to the polarization and activation of M1 macrophages, which is important for the engulfment and removal of apoptotic and necrotic cells (including cardiomyocytes and endothelial cells) during the early stage of I/R.^[^ ^21^ ^]^ Western Blot and ELISA were used to detect expression of MMP9, TGF‐*β*, VEGFA, Mertk, IL‐1*β*, IL‐6, and TNF‐*α* in protein levels (Figure 3Q–X), and the results showed a trend consistent with the RT‐qPCR results.

To determine the extent and importance of the regulatory effect of M2_EV_ on the recruitment of peripheral blood monocyte‐derived CCR2^+^ macrophages. Diphtheria toxin (DT) was administered to CCR2^DTR‐GFP^ mice to efficiently ablate peripheral blood CCR2^+^ monocytes and cardiac tissue resident CCR2^+^ macrophages. Four days post‐DT injection, CCR2^+^ monocytes and macrophages reappeared as circulating cells, indicating their rapid renewability (Figure S4D,E, Supporting Information). In the absence of cardiac tissue resident CCR2^+^ macrophages, the capacity of M2_EV_ to attenuate monocyte‐derived CCR2^+^ macrophages was markedly reduced (Figure S4F,G, Supporting Information), which suggested that M2_EV_ may exert their action through inhibiting inflammatory cascades that manifest in CCR2^+^ tissue‐resident macrophages. In addition, Lyz2^Cre/+^ R26^tdTomato/+^ (Lyz2 promoter targets myeloid lineage cells, mostly macrophages) was used to confirm the regulatory effect of M2_EV_ on the infiltration of myeloid monocytes. The results showed that M2_EV_ significantly inhibited the cardiac infiltration of proinflammatory monocytes (Ly6C^High^ Lyz2^tdtomato+^) and increased the cardiac infiltration of monocytes (Ly6C^low^ Lyz2^tdTomato+^) (Figure S4H,I, Supporting Information), which suggested the availability of these Ly6C^low^ Lyz2^tdTomato+^ monocytes to undergo transformation into reparative macrophage phenotypes.

It is well established that an acute inflammatory response contributes to cardiac remodeling and restoration of function following I/R, whereas excessive or uncontrollable inflammatory reactions lead to maladaptive functional cardiac remodeling in the long term.^[^ ^21^ ^]^ As previously noted, M1_EV_ improved infarct size but simultaneously promoted inflammation, 72 h after I/R. Therefore, we extended the endpoint of the experiment to explore the effects of M1_EV_ and M2_EV_ on long‐term heart function and cardiac remodeling after I/R, 14 days after I/R (Figure S5, Supporting Information). Cardiac ultrasound results show that the benefits of M1_EV_ were short‐lived, demonstrating an acceleration in the deterioration of cardiac function (Figure S5A,B, Supporting Information). H&E, Sirius Red, and multiple fluorescent staining for markers showed that M1_EV_ promoted myocardial necrosis, inflammatory cell infiltration, and collagen deposition (Figure S5C,D,G, Supporting Information). Flow cytometry analysis showed that M1_EV_ significantly increased the number of pro‐inflammatory (CD68^+^ IL‐1*β* ^+^) macrophages (Figure S5E,F, Supporting Information) and increased the level of inflammatory factors in the infarct border area (Figure S5H–J, Supporting Information). However, the beneficial outcomes of M2_EV_ were observable at 14 days after I/R, indicating an improved cardiac prognosis and an inhibition of early inflammation whilst initiating cardiac repair programs.

### 2.5 M2_EV_ Treatment Inhibits Excessive Glucose Uptake and mtROS Production by CCR2^+^ Macrophages

**Figure 4:**
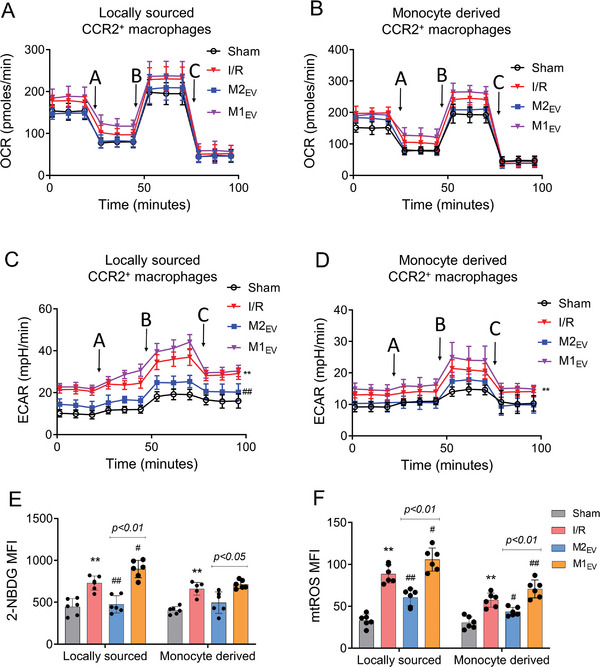
Mitochondrial respiration and glycolysis assays in CCR2^+^ macrophages. A,B) OCR and C,D) ECAR measurements in cardiac tissue‐resident and monocyte‐derived CCR2^+^ macrophages, as measured using a Seahorse Bioscience XF24 analyzer, *n* = 3, probed by the serial addition of A: oligomycin, B: FCCP, and C: antimycin A/rotenone as indicated. E) Glucose uptake in monocyte‐derived and locally resident CCR2^+^ macrophages measured using the fluorescence‐labeled glucose analogue (2‐NBDG) by mean fluorescence intensity (MFI), *n* = 6. F) mtROS levels in monocyte‐derived and locally resident CCR2^+^ macrophages using MitoSOX fluorescent probe, *n* = 6. All data are presented as the mean ± SD. Statistical significance is shown as ^**^p < 0.01 compared with the sham group and *^##^p* < 0.01 compared with the I/R group.

Macrophage polarization is closely tied to metabolic features, as demonstrated by the activation of proinflammatory macrophages or transition from M2 to M1 proinflammatory states exhibiting concurrent shifts from oxidative phosphorylation to lactate metabolism.^[^ ^22^ ^]^ We sought to explore whether ameliorated CCR2^+^ phenotypes after M2_EV_ treatment also regulated macrophage metabolic features, by utilizing Seahorse assays on two sources of CCR2^+^ macrophages. Mitochondrial respiratory resolution (OCR) and glycolysis (ECAR) in CCR2^+^ macrophages were detected, and results showed that after I/R injury, monocyte‐derived and cardiac tissue resident CCR2^+^ macrophages tended to exhibit statistically non‐significant increases in OCR (**Figure** **4**A,B). Respiration changes in CCR2^+^ macrophages were determined by FCCP‐induced uncoupling and subsequently, CCR2^+^ macrophages from both sources showed heightened efficiency in oxygen use. This result indicated that CCR2^+^ macrophages, regardless of source, exhibit higher respiratory reserve capacity to cope with the metabolic substrate load post‐I/R injury. Administration of M2_EV_ resulted in statistically non‐significant OCR reductions in CCR2^+^ macrophages from both sources. M1_EV_ tended to increase the OCR of I/R, but the results had no statistical significance. In parallel, both sources of CCR2^+^ macrophages demonstrated significantly up‐regulated glycolytic flux and lactic acid production, which suggested a requirement for increased uptake and utilization of glucose (Figure 4C,D), and these were further up‐regulated by M1_EV_ treatment. Administration of M2_EV_ tended to reduce ECAR readings in CCR2^+^ macrophages from both sources, but significant decreases were only calculated in locally sourced (tissue‐resident) CCR2^+^ macrophages. We next assessed CCR2^+^ macrophage utilization of glucose following I/R and M2_EV_ treatment. After I/R injury, the glucose uptake of CCR2^+^ macrophages from both sources was significantly enhanced. M2_EV_ inhibited the increased glucose uptake but M1_EV_ exacerbated the glucose uptake, showing statistical significance in only the cardiac tissue resident CCR2^+^ macrophages (Figure 4E). Increased glucose uptake by macrophages contributes to the production of excessive reactive oxygen species (ROS),^[^ ^23^ ^]^ and elevated ROS production in macrophages is more conducive to the polarization of macrophages to M1 inflammatory phenotypes.^[^ ^23^ ^]^ Thus, we measured mitochondrial ROS (mtROS) production using a mitochondrial‐targeted MitoSOX probe. After I/R injury, mtROS levels in CCR2^+^ macrophages from both sources increased significantly. M2_EV_ significantly attenuated the mtROS levels, whereas M1_EV_ significantly increased the mtROS level (Figure 4F).

### 2.6 M2_EV_ Treatment Promotes Myocardial Neovascularization

**Figure 5:**
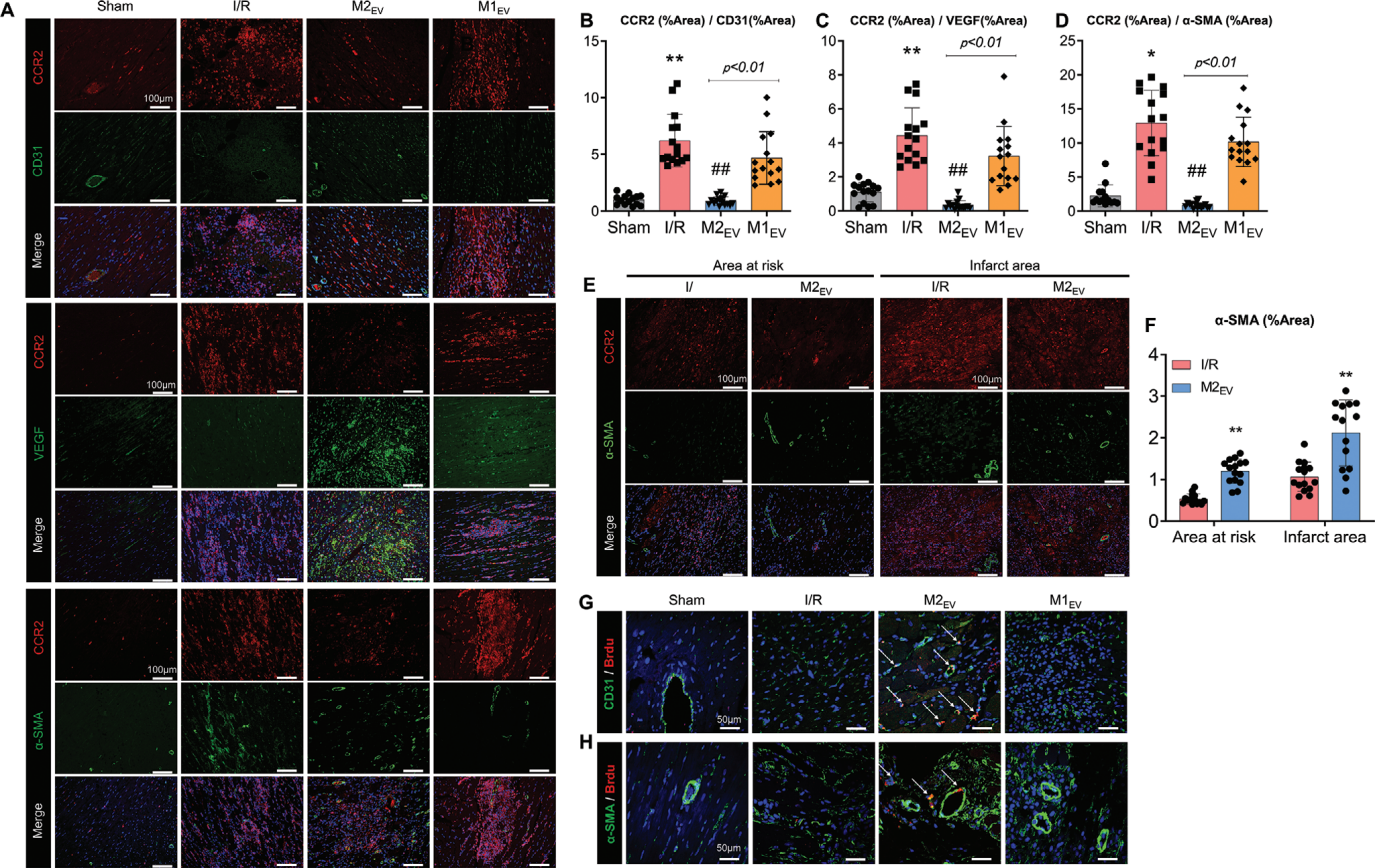
M2_EV_ inhibition of the CCR2 macrophage population promotes angiogenesis in rat and porcine myocardial I/R injury models. Co‐immunostaining of A) CCR2/CD31, CCR2/VEGF, and CCR2/*α*‐SMA in tissue sections of the infarction border zone of rat hearts. B–D) Quantitative analysis of CCR2: marker ratios from stained tissue sections in A (*n* = 3 rats in each group, 3–5 microscopic fields of view for each sample, scale bar = 100 µm). E) Immunostaining of *α*‐SMA in area at the risk and infarct areas of porcine hearts. F) Quantitative analysis of *α*‐SMA: marker ratios from stained tissue sections in E (*n* = 3 pigs per group, 3–5 microscopic fields of view for each sample, scale bar = 100 µm). Co‐immunostaining of G) CD31/BrdU and H) *α*‐SMA/BrdU in tissue sections of the infarction border zone of rat hearts (*n* = 3 rats in each group, scale bar = 50 µm, white arrows indicate positively co‐stained cells). Data presented as the mean ± SD. Statistical significance is shown as *p < 0.05 and ^**^ *p* < 0.01 compared with the control group; ^#^ *p* < 0.05 and ^##^ *p* < 0.01 compared with the I/R group.

In light of our observations that M2_EV_ inhibited the accumulation of CCR2^+^ macrophages and promoted the expression of tissue repair and ECM remodeling‐associated genes (VEGFA, TGF‐*β*, MMP9), we speculated that amelioration of I/R injury was related to the promotion of blood vessels formation and revascularization of the myocardium. Co‐immunostaining of rat cardiac tissue sections was performed to visualize CCR2 and endothelial cell marker, CD31; pro‐angiogenic cytokine, VEGF; or vascular smooth muscle cell marker, *α*‐smooth muscle actin (*α*‐SMA) (**Figure** **5**A). Quantification of the ratios of CCR2^+^ cells to each of the markers demonstrated that within the infarct border zone, M2_EV_ treatment significantly inhibited CCR2^+^ cell presence whilst increasing CD31^+^ cell numbers (Figure 5B) and VEGF production (Figure 5C). The expression of vasculature‐associated *α*‐SMA (tight circular staining pattern) increased following M2_EV_ therapy, but the expression of non‐vasculature‐associated *α*‐SMA correlated with the increase in CCR2, which explained the lesser changes in the quantification of the CCR2: *α*‐SMA ratio in treatment groups, compared to the sham group (Figure 5D). This suggested an increase in *α*‐SMA^+^ myofibroblast cell numbers in the I/R group. There were no significant differences between the M1_EV_ treatment and the I/R group in terms of CD31^+^ cell numbers, VEGF production, and the CCR2: *α*‐SMA expression ratio. Immunostaining in porcine cardiac tissue sections of the area at risk and infarct area reciprocated findings from the porcine models (Figure 5E,F). M2_EV_ treatment inhibited CCR2^+^ macrophage manifestation and promoted angiogenesis, as evidenced by increased detection of *α*‐SMA^+^ microvasculature. I/R injury resulted in increased non‐vasculature associated *α*‐SMA^+^ cells within the infarct area, which indicated the presence of myofibroblasts. In addition, immunofluorescent staining showed that M2_EV_ treatment increased BrdU staining, which co‐localized with DAPI staining of cell nuclei in CD31^+^ and *α*‐SMA^+^ vascular cells, indicative of neovascularization (Figure 5G,H).

We next assessed the capacity of human coronary artery endothelial cells (HCAEC) to migrate in vitro whilst co‐cultured with differentially treated BMDM (Figure S6A, Supporting Information). HCAEC co‐cultured with M2_EV_‐treated BMDM demonstrated enhanced migratory ability, compared to control cells. LPS+IFN*γ* stimulated BMDM (M1 phenotype) inhibited HCAEC migration and the addition of M2_EV_ (M1 to M2 switching) rescued the enhanced migratory ability. The addition of M2_EV_ recapitulated the enhanced migration exhibited by IL4‐treated HCAEC positive control experiments (Figure S6B, Supporting Information). Subsequent fixation and co‐staining of HCAEC for CD31 and VEGF indicated that LPS+IFN*γ* stimulated BMDM abolished the expression of VEGF, whereas the addition of M2_EV_ restored VEGF expression, to a similar staining intensity as observed in the IL4 positive control treated group (Figure S6C, Supporting Information). These data suggested that M2_EV_‐dependent polarization of macrophages toward the M2 phenotype facilitated the enhanced migration and pro‐angiogenic VEGF expression by endothelial cells, which explained the improved angiogenesis observed in cardiac tissue sections from M2_EV_‐treated myocardial I/R injury models.

### 2.7 MicroRNA‐181‐5p is a Candidate Effector for CCR2^+^ Macrophage Inhibition by M2_EV_

**Figure 6:**
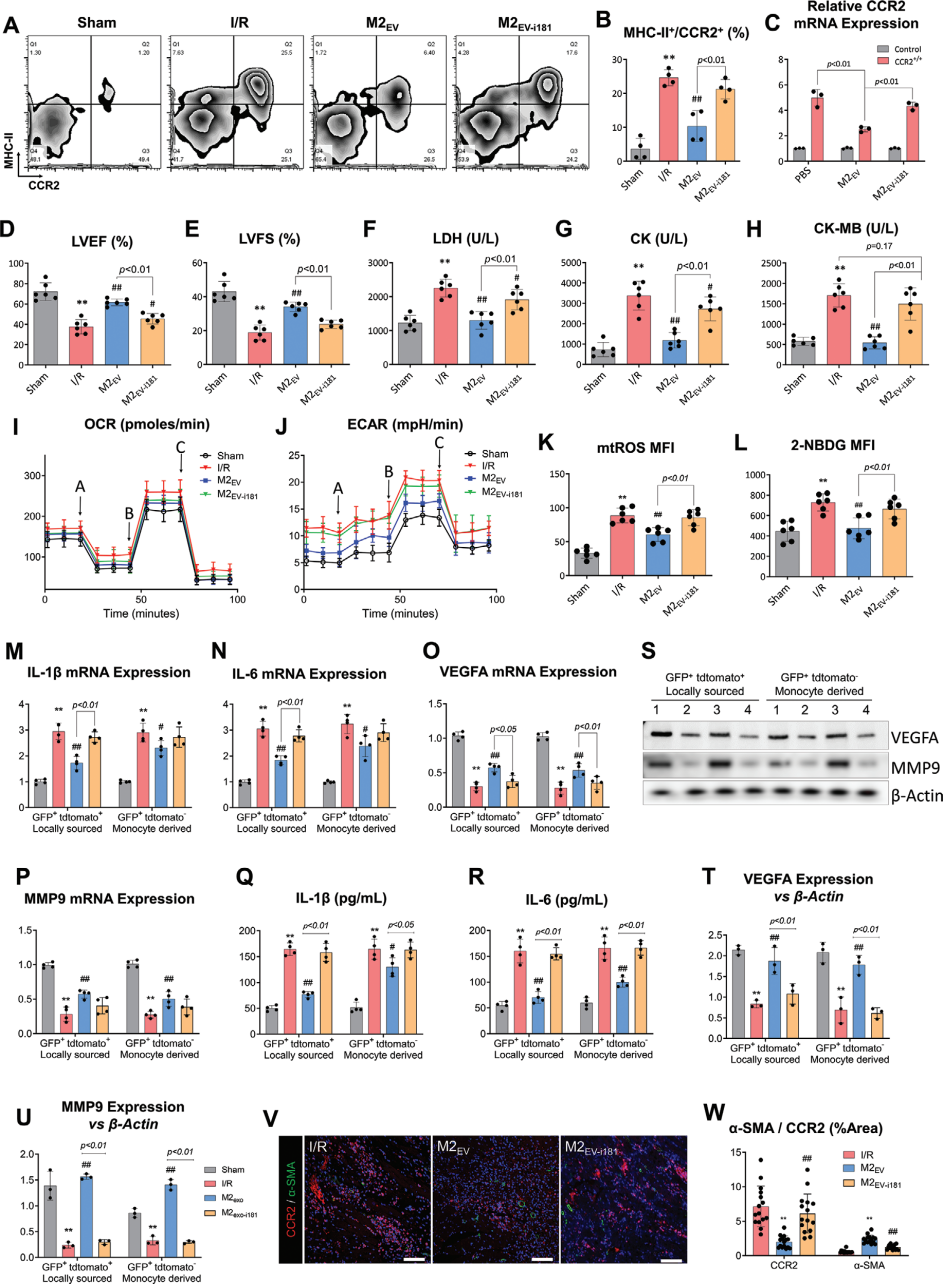
MicroRNA‐181b‐5p is implicated in CCR2 regulation by M2_EV_. A) Flow cytometry analysis of MHC‐II^Hi^/CCR2^−^ and MHC‐II^Hi^/CCR2^+^ cells in the infarct border zone tissue of rat I/R injured hearts. B) Quantified changes in MHC‐II^Hi^/CCR2^+^ cell populations under each treatment condition, *n* = 4. C) CCR2 mRNA expression in CCR2‐lentivirus infected BMDM incubated with M2_EV_ or M2_EV‐i181_, *n* = 3. D,E) Quantification of LVEF and LVFS, *n* = 6. F–H) Levels of LDH, CK, and CK‐MB in blood serum, *n* = 6. I,J) OCR and ECAR measurements in CCR2^+^ macrophages, as assessed by a Seahorse Bioscience XF24 analyzer, *n* = 3, probed by the serial addition of A: oligomycin, B: FCCP, and C: antimycin A/rotenone as indicated. K) Quantified mtROS MFI in CCR2^+^ macrophages, *n* = 6. L) Glucose uptake in CCR2^+^ macrophages measured using the fluorescence‐labeled glucose analogue, 2‐NBDG, *n* = 6. M–P) Phenotyping of resident and monocyte‐derived cardiac CCR2^+^ macrophages with M2_EV_ or M2_EV‐i181_, *n* = 4. Q,R) The protein levels of IL‐1*β* and IL‐6 in tissue‐resident and monocyte‐derived cardiac CCR2^+^ macrophages, as measured using ELISA, *n* = 4. S–U) The protein levels of MMP9 and VEGFA in tissue‐resident and monocyte‐derived cardiac CCR2^+^ macrophages, as detected using Western blot, *n* = 3. V) Co‐immunostaining of CCR2 and *α*‐SMA, and Sirius Red staining of collagen fibrils. Scale bar = 100 µm. W) Quantitative analysis of *α*‐SMA and CCR2 from Q (three rats in each group, 3–5 microscopic fields of view for each rat, scale bar = 100 µm). Data are presented as the mean ± SD. Statistical significance is shown as *p < 0.05 and ^**^ *p* < 0.01 compared with the control group; ^#^ *p* < 0.05 and ^##^ *p* < 0.01 compared with the I/R group.

High‐throughput microRNA expression profiling was used to assess small non‐coding RNA content within M2_EV_ that could potentially convey functional activity. M1_EV_ were assessed in tandem to determine differentially expressed small non‐coding RNA. The results of the microarrays and subsequent arrangement of the data into a volcano plot revealed that 55 microRNAs were prominently expressed in M2_EV_ (Figure S7A, Supporting Information). Previously, mitochondrial function was shown to be regulated by miR‐181 expression.^[^ ^24^ ^]^ Therefore, from the microRNAs with the highest expression change, we selected miR‐181b as a candidate for follow‐up experiments. To delineate the role of miR‐181 in conveying M2_EV_ functionality, we used lentivirus to overexpress the complementary sequence for miR‐181b‐5p and to inhibit its activity (i181) in M2 macrophages. Thus, miR‐181b‐5p present in sEV obtained from i181‐lentivirus infected M2 macrophages (M2_EV‐i181_) would be bound to its complementary sequence and be effectively prevented from exerting epigenetic regulation on target mRNA transcription (Figure S6B, Supporting Information). RT‐PCR confirmed that M2_EV‐i181_ could not increase the expression of miR‐181b in the rat heart (Figure S6C, Supporting Information), and M2_EV‐i181_ could not increase the expression of miR‐181b in CCR2^+^ macrophages (Figure S6D, Supporting Information). Flow cytometry analysis was used to assess changes in CCR2^+^ cells isolated from the infarct border zone tissue of rat I/R injury models (**Figure** **6**A,B). I/R injury markedly upregulated MHC‐II^+^/CCR2^+^ cell content compared to sham hearts. M2_EV_ treatment significantly attenuated MHC‐II^+^/CCR2^+^ cell counts, whereas M2_EV‐i181_ treatment failed to downregulate MHC‐II^+^/CCR2^+^ cells. This suggested that miR‐181b‐5p played a prominent role in regulating CCR2^+^ macrophages during myocardial injury. We confirmed the I/R injury induction of CCR2^+^ macrophages in vitro, by utilizing CCR2‐lentivirus to force the expression of CCR2 in BMDM. The CCR2‐overexpressing BMDM were treated with M2_EV_ or M2_EV‐i181_. The overexpression of CCR2 could be partially reversed by M2_EV_ treatment, but M2_EV‐i181_ failed to exhibit the same effect (Figure 6C).

The hearts of rats that received M2_EV‐i181_ treatment were assessed for functional parameters. Significantly lessened protective effects were observed in M2_EV‐i181_‐treated I/R hearts. LVEF and LVFS were significantly reduced compared to M2_EV_‐treated hearts (Figure 6D,E), whereas enzymes associated with myocardial injury (LDH, CK, CK‐MB) were detected at significantly higher concentrations in the M2_EV‐i181_ treatment group, compared to the M2_EV_ treatment group (Figure 6F–H). H&E staining showed that leukocyte infiltration was markedly increased in the M2_EV‐i181_ treatment group, compared to the M2_EV_ treatment group (Figure S8A, Supporting Information). The size of the infarcted area in I/R rats that received M2_EV‐i181_ was increased compared to I/R rats that received M2_EV_ (Figure S8B,C, Supporting Information). We next isolated CCR2^+^ macrophages (F4/80^+^GFP^+^) populations from the infarction border zone tissue of CCR2^DTR‐GFP^ mice using FACS. Subsequent Seahorse assays showed that M2_EV_‐i181 modulated OCR to a similar degree as M2_EV_, whereas M2_EV_‐i181 failed to significantly improve glycolysis (ECAR) in CCR2^+^ macrophages (Figure 6I,J). Compared with M2_EV_ treatments, M2_EV_‐i181 treatment was unable to significantly reduce glucose uptake and mtROS levels in CCR2^+^ macrophages (Figure 6K,L). The mRNA expression of a panel of inflammation‐regulating chemokines (*Ccl2*, *Cxcl2*, *Ccl11*, *Ccl24*, *Il4*, *Cxcl12*, *Il10*) reflected the inability of M2_EV‐i181_ to induce the repair phenotype to pro‐inflammatory phenotype macrophage switching (Figure S8D, Supporting Information). The expression of inflammation and repair‐related genes in CCR2^+^ macrophages from two different sources also confirmed this result (Figure 6M–P). Western Blot and ELISA were used to detect the expression of MMP9 and VEGFA protein levels (Figure 6Q–U), confirming the trend observed in the corresponding RT‐PCR results. Similar conclusions were obtained in vitro; compared with M2_EV_ treatment, M2_EV‐i181_ administration failed to inhibit NF*κ*B nuclear translocation induced by LPS+IFN*γ* (Figure S9A, Supporting Information), resulting in a preference for iNOS^+^ macrophages over ARG1^+^ macrophages (Figure S9B, Supporting Information). Taken together, these data suggest that miR‐181b was an imperative component of M2_EV_ involved in the regulation of macrophage metabolic function and thus, polarization.

The extent of angiogenesis conducive to cardiac tissue repair was determined by CCR2 and *α*‐SMA co‐immunostaining and quantification (Figure 6V). M2_EV‐i181_ treatment significantly upregulated *α*‐SMA^+^ staining compared to the I/R group, but this was not restricted to vasculature‐associated *α*‐SMA as in the M2_EV_ treatment group (Figure 6W). CCR2 quantification showed that M2_EV‐i181_ did not attenuate CCR2^+^ staining, as expected (Figure 6W). In vitro migration further supported our findings, as HCAEC co‐culture with LPS+IFN*γ* and M2_EV‐i181_ stimulated BMDM had attenuated migration (Figure S10A, Supporting Information) and partially reduced VEGF, as detected by immunocytochemistry (Figure S10B, Supporting Information). These data suggested that inhibition of miR‐181b‐5p activity resulted in a lack of regulatory capacity of M2_EV_ over CCR2^+^ macrophages, which impacted angiogenic potential and I/R injury repair.

Previously, miR‐181‐5p was confirmed to target the 3ʹ UTR of STAT3 mRNA to attenuate its expression.^[^ ^25^ ^]^ Phosphorylated STAT3 was shown to translocate to mitochondria to regulate mitochondrial metabolism and mtROS generation.^[^ ^26^ ^]^ Therefore, we sought to detect the protein expression of STAT3 in CCR2^+^ macrophages. I/R injury markedly upregulated the protein expression of STAT3 compared to sham hearts. M2_EV_ treatment significantly attenuated the protein expression of STAT3, whereas M2_EV‐i181_ treatment failed to downregulate the protein expression of STAT3 (Figure 6E,F). Subsequent Seahorse assays showed that M2_EV_ reversed the up‐regulation of OCR and ECR induced by the STAT3 activator, colivelin, and to a similar degree as the STAT3 inhibitor, S3I‐201 (Figure 6G,H).

### 2.8 Inhibition of CCR2 Receptor Expression could Enhance the Effect of M2_EV_

In the above experiments, we observed that M2_EV_ inhibited the accumulation of CCR2^+^ macrophages and promoted pro‐reparative phenotypes. Additionally, M2_EV_ treatment was suggested to affect CCR2^+^ resident macrophages to inhibit induced inflammatory cascades, including the recruitment and derivation of CCR2^+^ monocytes. We next sought to evaluate the influence of M2_EV_ on I/R injury after forcing the low or high expression of cardiac CCR2. Thus, we forced the knockdown or overexpression of CCR2 expression in rat hearts using intramyocardially injected adeno‐associated virus serotype 9 (AAV9) expressing short‐hairpin RNA targeting CCR2 (AAV9‐CCR2^−/−^) or the CCR2 ORF (AAV9‐CCR2^+/+^), respectively. Echocardiography‐based analyses showed that knockdown or overexpression of CCR2 did not drastically alter experimental outcomes, but we observed that there tended to be exacerbated I/R injury and a lessened beneficial effect of M2_EV_ on cardiac function in AAV9‐CCR2^+/+^ groups (Figure S11A,B, Supporting Information). Serum enzyme levels (Figure S11C–E, Supporting Information), TTC staining (Figure S11F, Supporting Information), and infarct size (Figure S11G, Supporting Information) demonstrated that miR‐181‐containing M2_EV_ maintained their protective activity on cardiac function and against infarct size regardless of knockdown or overexpression of cardiac CCR2, and these effects were diminished with the functional blockade of miR‐181.

## 3 Discussion

Myocardial I/R injury initiation of the acute inflammatory response follows a sequence of events that begins with the activation of CCR2^+^ resident macrophages. Recent evidence suggests that tissue‐resident leukocytes dictate the recruitment of mobilized leukocytes from the peripheral blood to injured tissues.^[^ ^12b^ ^]^ In the present study, we demonstrated that an induced shift in the cardiac macrophage population from a CCR2^+^ pro‐inflammatory macrophage subset to a reparative macrophage subset served as a critical determinant of successful LV remodeling, the rescue of cardiac function, and limitation of infarct size, in the context of reperfusion after AMI. Our investigation offers further evidence that achievement of the pro‐inflammatory to pro‐repair switch in the myocardium is suggested to be reliant on cell‐cell communication mediated through sEV production by M2 macrophages and subsequent uptake by CCR2^+^ macrophages. M2‐derived sEV (M2_EV_) had important roles in regulating the local CCR2^+^ macrophage population and the downstream recruitment of circulating CCR2^+^ monocytes. We identified that miR‐181‐5p delivery by M2_EV_ played important roles in the suppressive action on CCR2^+^ macrophage numbers and improving postinfarct LV remodeling, this process originated from the M2_EV_ regulation of glucose metabolism by CCR2^+^ macrophages. Our data suggested that effective reductions in cardiac‐resident CCR2^+^ macrophage numbers shortly after injury was conducive to three beneficial actions: i) the effective reduction of CCR2^+^ macrophage numbers resident to the myocardium shortly after the injury point was conducive to preventing inflammation from the recruitment of CCR2^+^ circulating monocytes; ii) the inhibition of monocyte/macrophage glucose metabolism helped to promote transition of CCR2^+^ macrophages to reparative phenotypes; and iii) the provision of an anti‐inflammatory myocardial tissue microenvironment facilitated myocardial neovascularization and cardiac tissue repair.

The current understanding of the origin and function of macrophages is gradually expanding. Within the past decade, we have learned that most organs contain both tissue‐resident and monocyte‐derived macrophages, and these tissues include healthy hearts and arterial vasculature. In cardiovascular disease, the differences in the biological functions of tissue‐resident and circulating monocyte‐derived macrophages have attracted considerable attention.^[^ ^27^ ^]^ For example, steady‐state cardiac resident macrophages were reported to contribute to the maintenance of cardiovascular health and the homeostasis of the cardiac environment by scavenging depleted mitochondria.^[^ ^5^, ^28^ ^]^ However, when the heart is damaged, such as in acute or chronic myocardial ischemia, CCR2^+^ macrophages residing in the heart activate and contribute to myocardial disease pathogenesis.^[^ ^20^, ^29^ ^]^ Following myocardial I/R injury, CCR2^+^ resident cardiac macrophages positively regulate the recruitment, trans‐endothelial migration, and infiltration of circulating monocytes into the myocardium, through a recently described distinct mechanism involving myeloid differentiation primary response‐88 (MyD88)‐dependent pathway activation.^[^ ^12^, ^13^, ^30^ ^]^ The increased proportion of a CCR2^+^ macrophage population, bolstered by the recruitment of CCR2^+^ monocyte‐derived macrophages, maintains the high‐intensity inflammatory response leading to myocardial tissue damage, maladaptive infarct repair, and LV remodeling.^[^ ^12b^ ^]^ Therefore, given the physiology and beneficial versus harmful effects of macrophage subsets and their interactions, we sought a rational design of a therapy that exploits macrophage cell‐cell communication to target the pro‐inflammatory/aggravating macrophage subsets and to improve acute myocardial infarction.

Traditionally defined M2 macrophages have been shown to inhibit inflammation and secrete factors and proteins related to tissue repair.^[^ ^27a^ ^]^ Cell‐derived sEV exhibit membrane and encapsulated contents similar in expression profile to their cellular origin.^[^ ^31^ ^]^ Thus, we selected M2_EV_ as a cell‐free therapeutic to regulate cardiac CCR2^+^ macrophages. The cardioprotective effects of M2_EV_ in rat and porcine models of I/R injury were dependent on preventing cardiac residence and accumulation of circulating monocyte‐derived CCR2^+^ macrophages. Following experiments wherein we cleared cardiac CCR2^+^ macrophages, the capability of M2_EV_ to inhibit monocyte‐derived CCR2^+^ macrophage accumulation significantly decreased, but the effects did not completely disappear. These results suggested that M2_EV_ primarily exerted their effects by acting on cardiac tissue resident CCR2^+^ macrophages to inhibit inflammation cascades that result in pro‐inflammatory monocyte infiltration.^[^ ^7c^ ^]^ However, other parallel mechanisms that potentially contributed to the observed M2_EV_ therapeutic effects, such as inhibition of vascular endothelial cell injury^[^ ^7c^ ^]^ and DAMPs release^[^ ^7^ ^]^ were not explored as part of this study. In the early stages of post‐I/R injury, we showed that CCR2^+^ macrophages from monocyte and myocardial sources showed typical M1 macrophage characteristics. In contrast, in the chronic phase of infarction, tissue‐resident, and monocyte‐derived macrophages were described to diverge from the canonical M1/M2 macrophage polarization pattern.^[^ ^32^ ^]^ A possible explanation for this difference may be that the macrophages of different infarct stages have differential characteristics, or through only assessing tissue‐resident or monocyte‐derived CCR2^+^ macrophages, we missed a separate upregulated macrophage subset with M2 similarities. Moreover, CCR2^+^ macrophages within the post‐infarct chronic phase regulate the expression of pro‐fibrotic genes in cells that govern cardiac fibrosis,^[^ ^33^ ^]^ which suggests that prolonged CCR2^+^ macrophage aggregation may improve the dynamic remodeling properties of the heart by regulating ECM secretion by cardiac fibroblasts to ultimately achieve amelioration of LV remodeling and cardiac dysfunction.^[^ ^31^ ^]^ In our study, M2_EV_ had the capacity to transform CCR2^+^ macrophages from pro‐inflammatory to pro‐reparative phenotypes, suggesting that early intervention by M2_EV_ promoted CCR2^+^ macrophages to switch to biological functions of cardiac repair. However, we observed that M2_EV_ also reduced CCR2^+^ macrophage accumulation, thereby also restricting a potential pool of pro‐reparative macrophage phenotypes. This contradictory action highlights deeper regulatory mechanisms of macrophages in cardiac repair that still require further clarification.

The activation and polarization of pro‐inflammatory macrophages are closely related to their metabolic mode, and more specifically, an energy supply mode reliant on glycolysis.^[^ ^34^ ^]^ Due to inhibited oxidative phosphorylation, the production of mtROS increases, which in turn promotes transcriptional expression and release of inflammatory factors.^[^ ^35^ ^]^ Our study showed an elevated glycolytic response in CCR2^+^ macrophages, irrespective of their tissue‐resident or monocyte‐derived origins. High glucose uptake results in the metabolic capacity of macrophages transitioning to the faster ATP synthesis of the lactate metabolism pathway, of which mtROS are byproducts.^[^ ^35^ ^]^ We showed that the mitochondrial aerobic respiration level of CCR2^+^ macrophages was increased, and this finding was consistent with the changes in mitochondrial metabolism shown by monocytes in patients with coronary heart disease.^[^ ^23^ ^]^ The observed changes may be adaptive responses to the lack of metabolic substrates within ischemic tissue microenvironments, and together with accumulating evidence, suggest that glucose intake should be carefully monitored in patients with cardiovascular diseases such as coronary heart disease. Here, we showed that M2_EV_ treatment resulted in the inhibition of glucose uptake by CCR2^+^ macrophages and reduced their glycolytic activity levels. These data, in part, explain M2_EV_ inhibition of inflammatory initiation of CCR2^+^ macrophages and metabolic mechanisms that promote macrophage switching to pro‐reparative phenotypes.

MicroRNA microarray data identified a key miRNA component that helped to partially explain how M2_EV_ conferred functional benefits. In this study, we found a total of 55 significantly up‐regulated miRNAs (compared with M1_EV_), which included miR‐181b and miR‐181d. Previously, miR‐181b was confirmed to regulate mitochondrial energy metabolism.^[^ ^24^ ^]^ We explored the roles that miR‐181b played in the therapeutic actions of M2_EV_ regulation over the metabolic mode of CCR2^+^ macrophages and the subsequent capacity to inhibit inflammatory responses. Expression of miR‐181b‐5p was implicated in a number of important cellular processes, including attenuation of pro‐inflammatory NF*κ*B signaling in endothelial cells, suppression of myeloid differentiation in acute myeloid leukemia, and mediation of hypoxia‐driven angiogenesis.^[^ ^36^ ^]^ In the present study, delivery of miR‐181b‐5p was imperative in lowering glucose uptake, shifting metabolic function toward lowered glycolytic activity, and reducing mtROS production in macrophages. Functional blockade of M2_EV_‐encapsulated miR‐181b‐5p activity attenuated improvements of glucose metabolism in CCR2^+^ macrophages, weakened the cardioprotective effect, and re‐enabled the recruitment and infiltration of CCR2^+^ monocytes to the infarct boundary area. Although our data suggest that miR‐181b‐5p had functional efficacy in reducing infarct size and regulating CCR2^+^ macrophage polarization, M2_EV_ also contains many other microRNAs, proteins, and cytokines. Undoubtedly, many of these other ingredients convey regulatory biological activities that may exert overall holistic functional benefits. Therefore, other M2_EV_‐encapsulated components may explain enhanced neo‐angiogenesis and infarct area recovery, and this line of thought warrants investigation in future studies utilizing M2_EV_.

In this study, we selected intramyocardial injection as an administration route to restrict the main focal point of the study to the myocardial interaction between sEV, resident macrophages, and roles in recruiting circulating monocytes. The intravenous injection may have resulted in a significant loss of sEV dose to liver and lung retention.^[^ ^37^ ^]^ Artificial reconstruction of sEV‐like nanoscale vesicles has shown promise as a strategy to improve myocardial uptake and reduce undesirable lung uptake following intravenous injection.^[^ ^38^ ^]^ Understandably, intramyocardial injection is an invasive procedure with inherent risks that limit this administration route in clinical practice. For therapeutic evaluation of M2_EV_ and a proof‐of‐concept study, intramyocardial injection served the purpose. However, in efforts to limit invasiveness, intrapericardial injection has emerged as a more innocuous choice.^[^ ^39^ ^]^ Furthermore, the prolonged and sustained release of M2_EV_ could be realized through incorporation into intrapericardially injected hydrogels, as demonstrated in recently developed exosome‐eluting pericardial hydrogel patches.^[^ ^40^ ^]^ These strategies, together with further delineation of the distinct similarities and differences between human haematopoietically derived macrophages and resident cardiac macrophages, open avenues of investigation that could help realize the progression of M2_EV_ to feasible clinical application.

Therapies based on sEV represent an important cell‐free alternative to cell transplant‐orientated therapies.^[^ ^31^, ^41^ ^]^ In the context of myocardial repair after AMI, the predominant beneficial effects of cell therapies were revealed to be the induction of an acute inflammatory response and a temporary stimulation of intrinsic wound‐healing cascades, rather than paracrine supplement by transplanted cells.^[^ ^33^ ^]^ This revelation highlighted the lack of mechanistic research on cell therapies, despite widespread use in clinical trials.^[^ ^42^ ^]^ Therapies that utilize sEV offer a route to maximize the most prevalent mechanisms that initiate efficacious biological effects in cardiac repair, whilst circumventing issues relating to the transplant of live cells into pro‐inflammatory tissue microenvironments.^[^ ^18^ ^]^ However, the requirement for an autologous sEV source and sufficient exosome yield remain the key limitations in upscaling sEV‐based therapies. The experiments in this study utilized M2_EV_ isolated from allogeneic cell sources. The use of allogeneic cells can offer the advantages of acquisition from various sources and the potential to accomplish “off‐the‐shelf” availability but may also elicit immunogenic effects. Idealistically, autologous cells would be used to offer the best clinical safety, yet timely treatment is critical and would be hampered by the harvest and expansion of patient cells to yield therapeutic doses of M2_EV_. Indeed, the rapid and facile reconstruction of exosome‐like nanovesicles from harvested cells has been proposed and may offer a potential solution.^[^ ^38a^ ^]^ We acknowledge that the composition and precise targeting mechanisms of BMDM‐derived sEV may not be physiologically identical to sEV produced by cardiac tissue‐resident macrophages, and mechanistic comparisons remain to be determined. Nonetheless, we have demonstrated that BMDMs served as a suitable reservoir of therapeutic sEV. To achieve sufficient yields of autologous sEV in a clinical setting, it is technically feasible to isolate human monocytes from peripheral blood and promote macrophage differentiation and expansion ex vivo,^[^ ^43^ ^]^ and such methods would require a thorough evaluation prior to the translational application of M2_EV_.

## 4 Conclusion

The results of our study provide evidence for the role of M2_EV_ in polarizing cardiac‐resident CCR2^+^ macrophages to an M2‐like phenotype, resulting in reduced recruitment of circulating CCR2^+^ monocytes, and an improved myocardial tissue microenvironment for cardiac tissue repair after I/R injury. Specifically, we demonstrated that miR‐181b‐5p was a key regulatory component within M2_EV_ that conferred cardioprotection by targeting CCR2^+^ macrophages to induce a distinctive polarization state. These data contribute to the cardioprotective/cardioregenerative properties already attributed to M2_EV_ (anti‐fibrotic effects; reduction of cardiomyocyte death and hypertrophy; myocardial regeneration; improved cardiac function; and angiogenesis). Whilst we have implicated reduced CCR2 expression as one downstream contributor to M2_EV_‐mediated cardioprotection, other plausible bioactive and synergistic effectors within M2_EV_ remain to be investigated. Moreover, our investigation offers compelling evidence in favor of a cell‐free and feasibly autologous‐sourced therapeutic alternative to cell therapy for the treatment of I/R injury.

## 5 Experimental Section

### Animal Use and Study Approval

All animal and surgical procedures conformed to Directive 2010/63/EU issued by the European Parliament. All animals were handled according to the guidelines of the TCM Animal Research Committee (TCM‐LAEC2019105) of Tianjin University of Traditional Chinese Medicine. Sprague–Dawley (SD) male rats (200 ± 20 g) and C57BL6/J mice (20 ± 20 g) were provided by Beijing Vital River Laboratory Animal Technology (Beijing, China). CCR2^CreER^ mice were kindly provided by Professor Jinping Feng (Tianjin Chest Hospital, China). R26^tdtomato^ mice and CCR2^DTR‐GFP^ mice were provided by Shanghai Biomodel Organism Science & Technology Development (Shanghai, China). Lyz2^Cre^ mice were provided by GemPharmatech (Jiangsu, China). The animals were housed in a specific‐pathogen‐free (SPF) animal facility in the experimental animal center of Tianjin University of Traditional Chinese Medicine, under controlled temperature (22 ± 2 °C) and humidity (40 ± 5%) with a 12‐h light/dark cycle and received a standard pellet diet with continual access to water. Adult male Minnesota–Hormel pigs (30 ± 3 kg) were provided by Nongnong Beijing Biotechnology (Beijing, China). The pigs were housed in the experimental animal center of Tianjin University of Traditional Chinese Medicine and fed a standard diet.

### Cell Culture

Bone marrow‐derived macrophages (BMDM) were isolated from bone marrow as previously described.^[^ ^44^ ^]^ Briefly, bones from rats and mice (femur) or pigs (rib) were sterilized with 75% ethanol for 10 min and flushed with Iscove's modified Dulbecco's medium (IMDM) containing 2% sEV‐free fetal bovine serum (FBS), the flushed solution was then filtered through a sterile 70 µm filter. Red blood cells were lysed with red blood cell lysis buffer (Solarbio, Beijing, China) and the whole solution was centrifuged, and the cell pellet was resuspended in fresh IMDM (containing 10% sEV‐free FBS, 20 ng mL^−1^ GM‐CSF). Cell cultures were replenished with fresh IMDM every third day. The macrophages were obtained after 10 days. Polarization of macrophages toward M1 using 1 µg mL^−1^ LPS (Sigma‐Aldrich, Poole, UK) and 50 ng mL^−1^ IFN‐*γ* (Cloud‐Clone Corp, Houston, TX, USA) stimulation for 24 h. M2 polarization was achieved using 20 ng mL^−1^ active IL‐4 and active IL‐13 (Cloud‐Clone Corp) stimulation for 24 h.^[^ ^18^, ^44^ ^]^ Human coronary artery endothelial cells (HCAECs) were purchased from Lonza (Walkersville, MD, USA). RAW264.7 macrophages were purchased from ATCC (Manassas, VA, USA). All cells were cultured according to the distributor's recommendations. Briefly, HCAEC were cultured in basal medium supplemented with EGM‐2MV (Lonza, USA). RAW264.7 cells were cultured in RPMI‐1640 medium (Gibco) supplemented with 10% sEV‐free FBS.

### Isolation and Characterization of sEV

sEV were isolated by ultracentrifugation as previously described.^[^ ^45^ ^]^ Briefly, the conditioned medium obtained from cultured macrophages was centrifuged at 300× *g* for 10 min. The supernatant was collected and centrifuged at 16 500× *g* for 30 min, followed by the filtration of the supernatant through a sterile 0.22 µm pore filter. The filtered solution was ultracentrifuged at 120 000× *g* for 120 min. The resultant supernatant was aspirated and discarded, and the remaining EVs were harvested. The M2_EV_ were identified using western blotting with the marker proteins CD9 (20597‐1‐AP; Proteintech), CD63 (25682‐1‐AP; Proteintech), Alix (12422‐1‐AP; Proteintech), and Calnexin (10427‐2‐AP; Proteintech). The protein concentration in exosomes was determined using the bicinchoninic acid kit (Thermo Fisher Scientific, Waltham, MA, USA). EVs were assessed for hydrodynamic size distribution by nano‐flow cytometry (NanoFCM). The size and morphology of M2_EV_ were detected by transmission electron microscopy (TEM) analysis using a Hitachi HT7700 (Hitachi, Japan). Briefly, M2_EV_ pellets (10 µg) were fixed with 2.5% paraformaldehyde for 30 min. The EVs pellets were deposited onto formvar carbon‐coated EM grids (EMS; FCF200‐Cu) and exposed for 10 min to a dry environment. Then, grids were washed five times, 3 min each time, with PBS. Exosomes were stained with 2% uranyl acetate for 5 min and observed and photographed by TEM.

### Ischemia‐Reperfusion (I/R) Models

I/R models were prepared according to our previous studies,^[^ ^45^, ^46^ ^]^ the rats and mice were anesthetized with an intraperitoneal injection of 5% tribromoethanol (30 mg kg^−1^), and thoracotomies were performed at the fourth intercostal space to expose the heart. A 6‐0 silk suture was used to ligate the left anterior descending coronary artery (LAD). After 60 min, the suture was removed to allow for reperfusion. The sham group underwent the same procedure without ligation. 30 min after reperfusion, M2_EV_ (Rat: 150 µg in 100 µL PBS, Mice: 50 µg in 50 µL PBS) were administered via five‐point intramyocardial injection.^[^ ^18^, ^45^ ^]^ Heart echocardiography was used to ensure all experimental animals of the untreated and treated groups exhibited a similar ventricular wall motion and EF% (± 3%). Porcine I/R models were prepared according to established methods.^[^ ^47^ ^]^ Briefly, the animals were sedated with intramuscular tiletamine and zolazepam (6 mg kg^−1^ each, suspended in Dexdomitor). General anesthesia was induced by intravenous injection of 3–10 mg kg^−1^ of 2% pentobarbital and mechanical ventilation with 40% oxygen was performed via oral intubation. Anesthesia was maintained by a continuous intravenous infusion (4–18 mg kg^−1^ h^−1^) of pentobarbital, and continuously monitored for their reflexes, electrocardiography (ECG), and respiratory status. To induce I/R injury, pigs were provided general anesthesia and then a 4‐0 silk suture was used to ligate the LAD (at the second diagonal branch) for 60 min.^[^ ^18^ ^]^ Then sutures were removed to allow for reperfusion. After a period of 30 min reperfusion time, M2_EV_ (1.0 mg in 500 µL PBS) was administered via five‐point intramyocardial injection,^[^ ^18^ ^]^ All pigs were treated with defibrillation at about 15, 30, and 45 min.

### Uptake of M2_EV_ In Vitro and In Vivo

To assess M2_EV_ uptake by cardiomyocytes or macrophages in vitro, the exosomes were labeled with PKH26 with the manufacturer's protocol (Sigma, USA). After PKH26 staining, the M2_EV_ was washed with PBS and collected by ultracentrifugation (120 000× *g* for 120 min) at 4 °C. Finally, PKH26 labeled EVs were resuspended in PBS. RAW264.7 cells (3 × 10^6^ cells per ML) were cultured at 37 °C in a 5% CO_2_ incubator. When cells were grown to 80% confluency, the cells were replenished with medium supplemented with M2_EV_ (5 × 10^8^ M2_EV_), which were labeled with PKH26. After 3 h, cells were washed three times with PBS. After stained nuclei with DAPI, cells were finally observed and photographed by a microscope. The in vivo uptake of PKH26 labeled M2_EV_ (150 µg in 100 µL PBS) was assessed after the administration of M2_EV_ via five‐point intramyocardial injection. After 0, 6, 12, 24, 48, and 72 h, epifluorescence was detected and radiant efficiency of the hearts was calculated using an IVIS Kinetic imaging system (PerkinElmer, Waltham, MA, USA) as previously described.^[^ ^46^ ^]^

### Magnetic‐Activated Cell Sorting (MACS) and Fluorescence‐Activated Cell Sorting (FACS) Separation

For single‐cell suspensions, the Multi‐Tissue Dissociation Kit 2 (Miltenyi, #130‐110‐203, Germany) and gentleMACS Dissociator device (Miltenyi) were used to dissociate cells from adult rat or mice hearts. Rat CD68^+^ and mouse CD45^+^ cells were isolated and purified using an AutoMACS Pro Separator (Miltenyi). Mouse CCR2^+^ macrophages, mouse monocyte‐derived macrophages, and cardiac tissue‐resident macrophages were isolated and purified using a FACSAria II (BD) flow cytometer. The procedures and information for flow cytometry are detailed in Supporting Information.

### Mitochondrial Respiration and Glycolysis

Oxygen consumption rate (OCR) and extracellular acidification rate (ECAR) were assessed using a Seahorse XF24 analyzer (Seahorse Bioscience) as previously described.^[^ ^48^ ^]^ Briefly, macrophages were cultured in 24‐well plates followed by the sequential addition of 1 µM oligomycin, 1 µM FCCP, and 1 µM rotenone combined with 1 µM antimycin A. OCR and ECAR were calculated using the pre‐loaded Seahorse analyzer software.

### Measurement of Glucose Uptake

Macrophages were incubated in a glucose‐free RPMI medium containing 5 µM fluorescent d‐glucose analogue 2‐[N‐(7‐nitrobenz‐2‐oxa‐1,3‐diazol‐4‐yl) amino]‐2‐deoxy‐d‐glucose (Cayman Chemical) for 60 min at 37 °C. Fluorescent intensities were analyzed using Lionheart FX automated imaging system (Bio Tek, USA).

### Measurement of mtROS

The isolation of intact mitochondria from macrophages was performed according to previously described protocols^[^ ^48^ ^]^ and using a commercially available mitochondrial extraction kit according to the manufacturer's protocols (Solarbio, China). Isolated mitochondria were transferred to a 96‐well flat‐bottomed plate and intramitochondrial ROS level was measured using a 2′,7′‐dichlorofluorescein diacetate (DCFH‐DA) fluorescent probe detection kit (Thermo Fisher Scientific) and a Lionheart FX automated imaging system (Bio Tek, USA).

### Statistical Analysis

Data were analyzed using SAS statistical software (v9.4, SAS Institute, NC, USA). All quantitative data are presented as the mean ± SD. Two‐tailed Student's *t*‐tests were used for pairwise comparisons. Differences across multiple groups with one variable were compared using a one‐way analysis of variance (ANOVA) or if groups had multiple variables, a two‐way ANOVA was used. ANOVAs were followed by Student–Newman–Keuls post hoc tests for multiple comparisons when the normality and homogeneity of the variance assumptions were satisfied. A *p‐*value < 0.05 was considered statistically significant. All experimental *n* numbers are provided in the figure legends.

## Conflict of Interest

The authors declare no conflict of interest.

## Supporting information

Supporting information

## Data Availability

Research data are not shared.

## References

[1] G. W. Reed , J. E. Rossi , C. P. Cannon , Lancet 2017, 389, 197. 27502078 10.1016/S0140-6736(16)30677-8

[2] G. Niccoli , G. Scalone , A. Lerman , F. Crea , Eur. Heart J. 2016, 37, 1024. 26364289 10.1093/eurheartj/ehv484

[3] a) D. J. Kereiakes , Circulation 2003, 108, III22; 14605016 10.1161/01.CIR.0000086951.09881.51

[4] W. E. Boden , R. A. O'Rourke , K. K. Teo , P. M. Hartigan , D. J. Maron , W. J. Kostuk , M. Knudtson , M. Dada , P. Casperson , C. L. Harris , B. R. Chaitman , L. Shaw , G. Gosselin , S. Nawaz , L. M. Title , G. Gau , A. S. Blaustein , D. C. Booth , E. R. Bates , J. A. Spertus , D. S. Berman , G. B. Mancini , W. S. Weintraub , N. Engl. J. Med. 2007, 356, 1503. 17387127 10.1056/NEJMoa070829

[5] a) Z. Wang , Y. L. Lu , W. T. Zhao , J. Zhong , X. Lin , Z. Sun , Y. He , M. Chen , L. R. Zheng , Basic Res. Cardiol. 2020, 115, 8; 31897858 10.1007/s00395-019-0769-3

[6] C. Peet , A. Ivetic , D. I. Bromage , A. M. Shah , Cardiovasc. Res. 2020, 116, 1101. 31841135 10.1093/cvr/cvz336 PMC7177720

[7] a) J. W. Jukema , J. J. Verschuren , T. A. Ahmed , P. H. Quax , Nat. Rev. Cardiol. 2011, 9, 53; 21912414 10.1038/nrcardio.2011.132

[8] a) M. Horckmans , L. Ring , J. Duchene , D. Santovito , M. J. Schloss , M. Drechsler , C. Weber , O. Soehnlein , S. Steffens , Eur. Heart J. 2017, 38, hw002; 10.1093/eurheartj/ehw002 28158426

[9] S. Epelman , K. J. Lavine , A. E. Beaudin , D. K. Sojka , J. A. Carrero , B. Calderon , T. Brija , E. L. Gautier , S. Ivanov , A. T. Satpathy , J. D. Schilling , R. Schwendener , I. Sergin , B. Razani , E. C. Forsberg , W. M. Yokoyama , E. R. Unanue , M. Colonna , G. J. Randolph , D. L. Mann , Immunity 2014, 40, 91. 24439267 10.1016/j.immuni.2013.11.019 PMC3923301

[10] K. J. Lavine , S. Epelman , K. Uchida , K. J. Weber , C. G. Nichols , J. D. Schilling , D. M. Ornitz , G. J. Randolph , D. L. Mann , Proc. Natl. Acad. Sci. U. S. A. 2014, 111, 16029. 25349429 10.1073/pnas.1406508111 PMC4234568

[11] J. Leid , J. Carrelha , H. Boukarabila , S. Epelman , S. E. Jacobsen , K. J. Lavine , Circ. Res. 2016, 118, 1498. 27009605 10.1161/CIRCRESAHA.115.308270 PMC5567774

[12] a) O. Dewald , P. Zymek , K. Winkelmann , A. Koerting , G. Ren , T. Abou‐Khamis , L. H. Michael , B. J. Rollins , M. L. Entman , N. G. Frangogiannis , Circ. Res. 2005, 96, 881; 15774854 10.1161/01.RES.0000163017.13772.3a

[13] a) M. Nahrendorf , F. K. Swirski , E. Aikawa , L. Stangenberg , T. Wurdinger , J. L. Figueiredo , P. Libby , R. Weissleder , M. J. Pittet , J. Exp. Med. 2007, 204, 3037; 18025128 10.1084/jem.20070885 PMC2118517

[14] A. Falkenham , T. Myers , C. Wong , J. F. Legare , Cardiovasc. Pathol. 2016, 25, 390. 27327107 10.1016/j.carpath.2016.05.006

[15] a) L. Barile , T. Moccetti , E. Marbán , G. Vassalli , Eur. Heart J. 2017, 38, hw304; 10.1093/eurheartj/ehw304 27443883

[16] a) J. Yao , K. Huang , D. Zhu , T. Chen , Y. Jiang , J. Zhang , L. Mi , H. Xuan , S. Hu , J. Li , Y. Zhou , K. Cheng , ACS Nano 2021, 15, 11099. 34152126 10.1021/acsnano.1c00628

[17] Y. Dai , S. Wang , S. Chang , D. Ren , S. Shali , C. Li , H. Yang , Z. Huang , J. Ge , J. Mol. Cell. Cardiol. 2020, 142, 65. 32087217 10.1016/j.yjmcc.2020.02.007

[18] G. de Couto , R. Gallet , L. Cambier , E. Jaghatspanyan , N. Makkar , J. F. Dawkins , B. P. Berman , E. Marban , Circulation 2017, 136, 200. 28411247 10.1161/CIRCULATIONAHA.116.024590 PMC5505791

[19] J. A. Reiffel , S. R. Gambino , D. M. McCarthy , E. B. Leahey Jr. , JAMA, J. Am. Med. Assoc. 1978, 239, 122. 10.1001/jama.239.2.122 579371

[20] K. Jin , S. Gao , P. Yang , R. Guo , D. Li , Y. Zhang , X. Lu , G. Fan , X. Fan , Small Methods 2022, 6, 2100752. 10.1002/smtd.202100752 35023642

[21] X. Yan , A. Anzai , Y. Katsumata , T. Matsuhashi , K. Ito , J. Endo , T. Yamamoto , A. Takeshima , K. Shinmura , W. Shen , K. Fukuda , M. Sano , J. Mol. Cell. Cardiol. 2013, 62, 24. 23644221 10.1016/j.yjmcc.2013.04.023

[22] a) T. Kobayashi , D. Nguyen‐Tien , Y. Sorimachi , Y. Sugiura , T. Suzuki , H. Karyu , S. Shimabukuro‐Demoto , T. Uemura , T. Okamura , T. Taguchi , K. Ueki , N. Kato , N. Goda , N. Dohmae , K. Takubo , M. Suematsu , N. Toyama‐Sorimachi , Proc. Natl. Acad. Sci. U. S. A. 2021, 118, 2100295118. 10.1073/pnas.2100295118 PMC8379930 34385317

[23] T. Shirai , R. R. Nazarewicz , B. B. Wallis , R. E. Yanes , R. Watanabe , M. Hilhorst , L. Tian , D. G. Harrison , J. C. Giacomini , T. L. Assimes , J. J. Goronzy , C. M. Weyand , J. Exp. Med. 2016, 213, 337. 26926996 10.1084/jem.20150900 PMC4813677

[24] A. Indrieri , S. Carrella , A. Romano , A. Spaziano , E. Marrocco , E. Fernandez‐Vizarra , S. Barbato , M. Pizzo , Y. Ezhova , F. M. Golia , L. Ciampi , R. Tammaro , J. Henao‐Mejia , A. Williams , R. A. Flavell , E. De Leonibus , M. Zeviani , E. M. Surace , S. Banfi , B. Franco , EMBO Mol. Med. 2019, 11, 8734. 10.15252/emmm.201708734 PMC6505685 30979712

[25] Y. Qu , Q. Zhang , X. Cai , F. Li , Z. Ma , M. Xu , L. Lu , J. Cell. Mol. Med. 2017, 21, 2491. 28382720 10.1111/jcmm.13170 PMC5618698

[26] a) J. Wegrzyn , R. Potla , Y. J. Chwae , N. B. Sepuri , Q. Zhang , T. Koeck , M. Derecka , K. Szczepanek , M. Szelag , A. Gornicka , A. Moh , S. Moghaddas , Q. Chen , S. Bobbili , J. Cichy , J. Dulak , D. P. Baker , A. Wolfman , D. Stuehr , M. O. Hassan , X. Y. Fu , N. Avadhani , J. I. Drake , P. Fawcett , E. J. Lesnefsky , A. C. Larner , Science 2009, 323, 793; 19131594 10.1126/science.1164551 PMC2758306

[27] a) T. A. Wynn , A. Chawla , J. W. Pollard , Nature 2013, 496, 445; 23619691 10.1038/nature12034 PMC3725458

[28] J. A. Nicolás‐Ávila , A. V. Lechuga‐Vieco , L. Esteban‐Martínez , M. Sánchez‐Díaz , E. Díaz‐García , D. J. Santiago , A. Rubio‐Ponce , J. L. Li , A. Balachander , J. A. Quintana , R. Martínez‐de‐Mena , B. Castejón‐Vega , A. Pun‐García , P. G. Través , E. Bonzón‐Kulichenko , F. García‐Marqués , L. Cussó , A. G. N , A. González‐Guerra , M. Roche‐Molina , S. Martin‐Salamanca , G. Crainiciuc , G. Guzmán , J. Larrazabal , E. Herrero‐Galán , J. Alegre‐Cebollada , G. Lemke , C. V. Rothlin , L. J. Jimenez‐Borreguero , G. Reyes , et al., Cell 2020, 183, 94. 32937105 10.1016/j.cell.2020.08.031

[29] a) N. R. Wong , J. Mohan , B. J. Kopecky , S. Guo , L. Du , J. Leid , G. Feng , I. Lokshina , O. Dmytrenko , H. Luehmann , G. Bajpai , L. Ewald , L. Bell , N. Patel , A. Bredemeyer , C. J. Weinheimer , J. M. Nigro , A. Kovacs , S. Morimoto , P. O. Bayguinov , M. R. Fisher , W. T. Stump , M. Greenberg , J. A. J. Fitzpatrick , S. Epelman , D. Kreisel , R. Sah , Y. Liu , H. Hu , K. J. Lavine , Immunity 2021, 54, 2072; 34320366 10.1016/j.immuni.2021.07.003 PMC8446343

[30] a) B. Chen , A. Brickshawana , N. G. Frangogiannis , Circ. Res. 2019, 124, 183; 30653429 10.1161/CIRCRESAHA.118.314357 PMC6338423

[31] R. Kalluri , V. S. LeBleu , Science 2020, 367, aau6977. 10.1126/science.aau6977 PMC7717626 32029601

[32] H. B. Sager , M. Hulsmans , K. J. Lavine , M. B. Moreira , T. Heidt , G. Courties , Y. Sun , Y. Iwamoto , B. Tricot , O. F. Khan , J. E. Dahlman , A. Borodovsky , K. Fitzgerald , D. G. Anderson , R. Weissleder , P. Libby , F. K. Swirski , M. Nahrendorf , Circ. Res. 2016, 119, 853. 27444755 10.1161/CIRCRESAHA.116.309001 PMC5378496

[33] R. J. Vagnozzi , M. Maillet , M. A. Sargent , H. Khalil , A. K. Z. Johansen , J. A. Schwanekamp , A. J. York , V. Huang , M. Nahrendorf , S. Sadayappan , J. D. Molkentin , Nature 2020, 577, 405. 31775156 10.1038/s41586-019-1802-2 PMC6962570

[34] a) O. R. Colegio , N. Q. Chu , A. L. Szabo , T. Chu , A. M. Rhebergen , V. Jairam , N. Cyrus , C. E. Brokowski , S. C. Eisenbarth , G. M. Phillips , G. W. Cline , A. J. Phillips , R. Medzhitov , Nature 2014, 513, 559; 25043024 10.1038/nature13490 PMC4301845

[35] C. Nathan , A. Cunningham‐Bussel , Nat. Rev. Immunol. 2013, 13, 349. 23618831 10.1038/nri3423 PMC4250048

[36] a) P. Hartmann , A. Schober , C. Weber , Cell. Mol. Life Sci. 2015, 72, 3253; 26001902 10.1007/s00018-015-1925-z PMC4531138

[37] M. Zhao , S. Liu , C. Wang , Y. Wang , M. Wan , F. Liu , M. Gong , Y. Yuan , Y. Chen , J. Cheng , Y. Lu , J. Liu , ACS Nano 2021, 15, 1519. 33369392 10.1021/acsnano.0c08947

[38] a) C. Qi , X. Liu , D. Zhi , Y. Tai , Y. Liu , Q. Sun , K. Wang , S. Wang , A. C. Midgley , D. Kong , Nano Res. 2022, 15, 1680;

[39] X. Mei , D. Zhu , J. Li , K. Huang , S. Hu , Z. Li , B. L. de Juan Abad , K. Cheng , Med 2021, 2, 1253. 34825239 10.1016/j.medj.2021.10.001 PMC8612456

[40] G. Cheng , D. Zhu , K. Huang , T. G. Caranasos , J. Mol. Cell. Cardiol. 2022, 169, 113. 35523270 10.1016/j.yjmcc.2022.04.020

[41] B. Yang , Y. Chen , J. Shi , Adv. Mater. 2019, 31, 1802896. 10.1002/adma.201802896 30126052

[42] a) J. A. Epstein , JAMA Cardiol. 2019, 4, 95; 30480702 10.1001/jamacardio.2018.4435

[43] F. J. Rios , R. M. Touyz , A. C. Montezano , Methods Mol. Biol. 2017, 1527, 311. 28116726 10.1007/978-1-4939-6625-7_24

[44] a) W. Ying , M. Riopel , G. Bandyopadhyay , Y. Dong , A. Birmingham , J. B. Seo , J. M. Ofrecio , J. Wollam , A. Hernandez‐Carretero , W. Fu , P. Li , J. M. Olefsky , Cell 2017, 171, 372; 28942920 10.1016/j.cell.2017.08.035

[45] Y. Zou , L. Li , Y. Li , S. Chen , X. Xie , X. Jin , X. Wang , C. Ma , G. Fan , W. Wang , ACS Appl. Mater. Interfaces 2021, 13, 56892. 34823355 10.1021/acsami.1c16481

[46] L. Li , Y. Wang , R. Guo , S. Li , J. Ni , S. Gao , X. Gao , J. Mao , Y. Zhu , P. Wu , H. Wang , D. Kong , H. Zhang , M. Zhu , G. Fan , J. Controlled Release 2020, 317, 259. 10.1016/j.jconrel.2019.11.032 PMC7384207 31783047

[47] a) L. O. Karlsson , N. Bergh , L. Li , E. Bissessar , I. Bobrova , G. J. Gross , L. M. Akyürek , L. Grip , Eur. J. Pharmacol. 2012, 674, 378; 22119384 10.1016/j.ejphar.2011.11.012

[48] L. Li , J. Ni , M. Li , J. Chen , L. Han , Y. Zhu , D. Kong , J. Mao , Y. Wang , B. Zhang , M. Zhu , X. Gao , G. Fan , Drug Delivery 2017, 24, 1617. 29063791 10.1080/10717544.2017.1391893 PMC8241051

