# Supplementary material for: M2 Macrophage‐Derived sEV Regulate Pro‐Inflammatory CCR2+ Macrophage Subpopulations to Favor Post‐AMI Cardiac Repair
Source: Adv Sci (Weinh). 2023 Mar 22;10(14):2202964. doi: 10.1002/advs.202202964 (PMC10190454; doi:10.1002/advs.202202964)
Supplement: Supplementary file 1 — Supporting information [file ADVS-10-2202964-s001.pdf]

ADVANCED SCIENCE
Open Access

Supporting Information

for *Adv. Sci.*, DOI 10.1002/advs.202202964

M2 Macrophage-Derived sEV Regulate Pro-Inflammatory CCR2$^+$ Macrophage Subpopulations to Favor Post-AMI Cardiac Repair

Lan Li, Jiasong Cao, Sheng Li, Tianyi Cui, Jingyu Ni, Han Zhang, Yan Zhu, Jingyuan Mao, Xiumei Gao, Adam C. Midgley*, Meifeng Zhu* and Guanwei Fan**

Supplementary Materials for

Title: M2 macrophage-derived sEV regulate pro-inflammatory CCR2$^+$ macrophage subpopulations to favor post-AMI cardiac repair

One Sentence Summary: M2 macrophage-derived sEV ameliorate CCR2$^+$ macrophage-driven AMI.

Authors: Lan Li[1,2, †], Jiasong Cao [4, †], Sheng Li[1], Tianyi Cui[1], Jingyu Ni[1,2], Han Zhang[1], Yan Zhu[1], Jingyuan Mao[2], Xiumei Gao[1, *], Adam C. Midgley[3, *], Meifeng Zhu[3*], Guanwei Fan[1,2, *]

Affiliations:

[1]State Key Laboratory of Modern Chinese Medicine, Key Laboratory of Pharmacology of Traditional Chinese Medical Formulae, Ministry of Education, Tianjin University of Traditional Chinese Medicine, Tianjin, China.

[2]National Clinical Research Center for Chinese Medicine Acupuncture and Moxibustion, State Key Laboratory of Component-based Chinese Medicine, First Teaching Hospital of Tianjin University of Traditional Chinese Medicine; Tianjin, China.

[3]Key Laboratory of Bioactive Materials for the Ministry of Education, College of Life Sciences, Nankai University; Tianjin, China.

[4]Tianjin Key Laboratory of Human Development and Reproductive Regulation, Tianjin Central Hospital of Gynecology Obstetrics; Tianjin, China.

*Corresponding authors: Prof. X. Gao (gaoxiumei@tjutcm.edu.cn); Prof. A.C. Midgley (midgleyac@nankai.edu.cn); Prof. M. Zhu (zhumeifeng2013@126.com); Prof. G. Fan (guanwei.fan@tjutcm.edu.cn).

[†]Authors contributed equally to the work.

Supplementary Methods

Transfected and Transgenic animal procedures.: Specific CCR2 knock-down or overexpression was achieved in the left ventricles of the rats by using adeno-associated viruses-based (AAV9) delivery vectors (Genechem, Shanghai, China). In short, following tracheal intubation and thoracotomy, AAV9 expressing the CCR2 open reading frame (titer: 1.32×10^{12}), CCR2-shRNA (titer: 1.43×10^{12}), or a scrambled control sequence bearing no

homology to known gene transcripts (titer: 1.14×10^{12}) were injected into the left ventricle at multiple sites. After a period of 45 days, the rats were subjected to I/R surgery and subsequent treatments. Six-week-old $CCR2^{CreER/+}$ $R26^{tdtomato/+}$ mice received daily intraperitoneal injections of tamoxifen (2 mg) in corn oil, for 5 days. After three weeks, $CCR2^{CreER/+}$ $R26^{tdtomato/+}$ mice were prepared for surgery as described *above.* Seven/eight-week-old $CCR2^{DTR\text{-}GFP}$ mice received daily intraperitoneal injections of DT (50 μg/kg body weight), for 3 days. The following *day,* $CCR2^{DTR\text{-}GFP}$ mice were prepared for surgery as described *above.* Seven/eight-week-old $Lyz2^{Cre/+}$ $R26^{tdTomato/+}$ mice received daily intraperitoneal injections of tamoxifen (2 mg) in corn oil, for 5 days. After 7 days, $Lyz2^{Cre/+}$ $R26^{tdtomato/+}$ mice were prepared for surgery as described above.

Microarray-based high-throughput microRNA expression profiling: MicroRNA sequencing was performed using nucleic acids isolated from $M2_{EV}$ and $M1_{EV}$. Analysis and bioinformatics analysis was outsourced and performed by Ouyi Biomedical Technology (Shanghai, China). In short, the Agilent Rat miRNA Microarray Kit (Release 21.0, 8x15K, Design-ID: 070154) was used to evaluate 6 independent samples for the expression profiles of 780 mature miRNA. Total RNA was quantified by a NanoDrop ND-2000 (Thermo-Fisher Scientific) and the RNA integrity (RIN) was assessed using an Agilent Bioanalyzer 2100 (Agilent Technologies, USA). The sample labeling, microarray hybridization, and washing were performed based on the manufacturer’s standard protocols. Briefly, total RNA was dephosphorylated, denaturated, and then labeled with Cyanine-3-CTP. After purification the labeled RNAs were hybridized onto the microarray. After washing, the microarrays were scanned with an Agilent Scanner G2505C (Agilent Technologies). Feature Extraction software (v10.7.1.1, Agilent Technologies) was used to analyze array images to calculate raw data. Next, raw data was normalized using the quantile algorithm. The probes present in at least 100 % of samples in any 1 of the 2 conditions were flagged as "Detected" and were selected for further data analysis. Differentially expressed miRNAs were then identified through fold-change as well as P-values calculated by *t*-test. The thresholds set for up- and down-regulated genes were a fold-change ≥ 2.0 and a P-value ≤ 0.05.

Cardiac functional measurements: Rat echocardiography was performed using a Vevo 2100 (VisualSonic, Canada) ultra-high-resolution animal ultrasound imaging system and the left ventricular function was evaluated as previously described. Long axes with M-mode were visualized. Three representative cycles were captured for each animal, and measurements for ejection fraction (EF%), fractional shortening (FS%). Rat left ventricular pressure-volume

was determined using a Millar pressure-volume catheter (SPE-869, #840-813G) was connected to an MPVS Ultra PV loop system (Millar, Houston, TX, USA). After calibration, the catheter was advanced into the left ventricle through the right common carotid artery. The pressure-volume loop was recorded using a data acquisition system (LabChart Pro, ADInstruments, Colorado Springs, CO, USA) and measurements for dP/dt max and dP/dt were calculated. Pig echocardiography was performed using a Philips EPIQ 7C ultrasound system (Philips, Cambridge, MA, USA) and was used to evaluate the ejection fraction (EF%) before LAD and after 72 h of reperfusion. Electrocardiograms (ECG, Schiller, AT-102, Switzerland) were recorded before LAD and after 72 h of reperfusion.

Plasma biochemistry and enzyme-linked immunosorbent assay (ELISA): Plasma biochemistry, including creatine kinase (CK), creatine kinase-MB (CK-MB), and lactate dehydrogenase (LDH) in rats, Interleukin-1 beta (IL-1β) in mouse and rat, Interleukin-6 (IL-6) in mouse and rat, human tumor necrosis factor α (TNF-α) in mouse and rat, and cardiac troponin (CTn) in pig was assessed using an auto-analyzer (Multiskan MK3; Thermo-Fisher Scientific).

2,3,5-Triphenyltetrazolium chloride (TTC) staining: After 72 h of reperfusion, the rats and pigs were euthanized, hearts were excised, and were washed in PBS. Following harvest, rat hearts were cut into six transverse slices (from apex to basal edge of infarction), whereas pig hearts were sectioned into 1 cm thick slices (below the ligation line). Sections were incubated with 1% 2,3,5-triphenyltetrazolium chloride (TTC) in PBS for 30 min, at 37°C, and in darkness. Then, the transverse slices were washed with PBS three times and subsequently imaged. The infarcted myocardium was stained in white, and the viable tissue was stained in red. Photographs were taken and images were analyzed using ImageJ software (NIH, Bethesda, MD, USA). The percentage of infarct area was calculated using the following equation:

$$Infarct\ area\ (\%) = \frac{Infarct\ area}{Viable\ area + Infarct\ area} \times 100$$

Histological analysis: The heart tissues were fixed by immersion in 4% paraformaldehyde for 72 h. The tissues were then embedded in paraffin, cut into 4 μm thick sections, and subjected to hematoxylin/eosin staining (H&E). For immunofluorescence (IF) analysis, sections were incubated with CCR2 (Rat: Abcam, Rabbit Monoclonal, ab273050; Pig: Abcam, Rabbit Monoclonal, ab216863, 1:200 dilution), α-SMA (Proteintech, Mouse Monoclonal, 67735-1-Ig, 1:200 dilution), VEGF (Proteintech, Mouse Monoclonal, 66828-1-

Ig, 1:200 dilution), CD31 (Servicebio, Mouse Monoclonal, GB12063, 1:200 dilution), CD68 (Pig: Proteintech, Mouse Monoclonal, 66231-2-Ig, 1:200 dilution), or Brdu (Proteintech, Mouse Monoclonal, 66241-1-Ig) at 4 °C for 12 h and subsequently incubated with anti-Rabbit HRP (Alexa Fluor 647) and anti-Mouse HRP (Alexa Fluor 488) at 37 °C for 2 h. After washing with PBS, the sections were counterstained with DAPI. The sections were examined using a fluorescence microscope and a digital camera (Carl Zeiss, Axio Observer Al, Germany). PKH26 labeled $M2_{EV}$ and CCR2 colocalization analysis on cryosections. The tyramide signal amplification plus multiplex fluorescence staining kit (#G1236-100T, Servicebio, Wuhan, China) was used for staining CCR2 (Abcam, Rabbit Monoclonal, ab273050), CD68 (Proteintech, Rabbit Monoclonal, 25747-1-AP), IL-1β (Abcam, Rabbit Monoclonal, ab283818) / ARG1 (Proteintech, Mouse Monoclonal, 66129-1-Ig) or Collagen Hybridizing Peptide (CHP, 3HeliX, RED300), according to the manufacturer's protocol. The sections were examined and imaged using a NanoZoomer Digital Pathology (NanoZoomer S60, Japan, DAPI: ultraviolet 330-380nm, emission 420nm, FITC: ultraviolet 465-495nm, emission 515-555nm; Cy3: ultraviolet 510-560 nm, emission 590 nm; Cy5: ultraviolet 608-648nm, emission 672-712 nm).

Magnetic-activated cell sorting (MACS) and fluorescence-activated cell sorting (FACS) separation： For single-cell suspensions, the Multi-Tissue Dissociation Kit 2 (Miltenyi, #130-110-203, Germany) was used to dissociate cells from adult rat or mice hearts according to the distributor's recommendations. Briefly, heart tissues harvested from rats and mice were cut at the infarction border zone (~2 mm in width around the infarcted myocardium) into small pieces (1-2 mm^3) and suspended in 2.5 mL of enzyme mix in gentleMACS C-Tubes (Miltenyi, #130-093-237). The C-Tubes were attached onto the sleeve of a gentleMACS Dissociator device (Miltenyi), and the program "37C_Multi_G" was run. Samples were then left to settle without agitation for 15 min at 37 °C before the program was repeated, or until the tissues sections has disassociated completely. Following erythrocyte removal, Rat $CD68^+$ cells (anti-CD68 antibody, Miltenyi, #130-102-723; Anti-FITC MicroBeads, Miltenyi, # 130-048-701) were isolated and purified using an AutoMACS Pro Separator (Miltenyi). Mouse $CD45^+$ cells were isolated and purified using EasySep™ Mouse CD45 Positive Selection Kit (Stemcell, # 18945). Mouse monocyte derived macrophages (GFP^+ $tdTomato^-$) and locally resident macrophages (GFP^+ $tdTomato^+$) were isolated and purified from $CCR2^{CreER/+}$ $R26^{tdTomato/+}$ mice by fluorescence-activated cell sorting (FACS). Antibody List: TruStain FcX™ (anti-mouse CD16/32) Antibody (Biolegend, # 101319), APC/Cyanine7 anti-mouse

CD45 Antibody (Biolegend, # 103116); BV650 Rat Anti-CD11b (BD Horizon™, # 563402); Brilliant Violet 421™ anti-mouse CD192 (CCR2) Antibody (Biolegend, # 150605); PerCP anti-mouse F4/80 Antibody (Biolegend, # 123126). The following antibodies were used for flow cytometric analysis: CD68 Antibody, anti-rat, PE, REAfinity™ (Miltenyi, # 130-123-757), Anti-CCR2 antibody conjugated to DyLight 488 (Invitrogen, #PA5-23040); anti-MHC Class II antibody conjugated to APC (Invitrogen, #17-0920-82), TruStain FcX™ (anti-mouse CD16/32) Antibody (Biolegend, # 101319); APC/Cyanine7 anti-mouse CD45 Antibody (Biolegend, # 103116), PE anti-mouse CD192 (CCR2) Antibody (Biolegend, # 150610), BV650 Rat Anti-CD11b (BD Horizon™, # 563402), Anti-IL-1 beta (abcam, # ab254360). The collected cells were incubated with antibodies at 37°C for 20 min. Flow cytometry was performed using a FACSAria II flow cytometer (BD Biosciences, USA) and a NovoCyte Flow Cytometers (Agilent). Data were analyzed with FlowJo software version V10.8 (TreeStar, USA) and NovoExpress (Agilent).

Cell transfection: For cell transfections, M2 macrophages were stably transfected with CCR2-overexpressing lentivirus (Genechem, Shanghai, China), miR-181b-5p inhibitory sequence (i181) containing lentivirus (Genechem), or lentivirus containing scrambled controls sequences. Briefly, M2 macrophages was inoculated into 6-well plate and cultured at 37°C. The infection reagent, and lentivirus were added to M2 macrophages according to the manufacturer's instructions for 72 h.

Quantitative real-time polymerase chain reaction (RT-PCR): The mRNA reverse transcription kit was used to obtain cDNA following the manufacturer's instructions (Roche, Basle, Switzerland). SYBR Green PCR master mix (Roche) was used to perform RT-PCR using a CFX96TM PCR detection system (BioRad, Redmond, WA, USA). GAPDH and U87 were used as a housekeeping gene. All primer sequences are provided in the supplementary data.

Table S1. Primer sequences information

Target gene	Primer sequence (5' → 3')	
Ccl2	Forward:	TCTCTTCCTCCACCACTATGC
	Reverse:	GTTCTCCAGCCGACTCATTG
Cxcl2	Forward:	AAAGATACTGAACAAAGGCAAGG
	Reverse:	CAGACAGCGAGGCACATC
Ccl11	Forward:	CTTCTATTCCTGCTGCTCAC
	Reverse:	GCTCTTCAGTAGTGTGTTGG

Ccl24	Forward:	TGTTTGTCTGTCCACCTCTG
	Reverse:	AAATGCTGCCGTTGAAATCC
Il4	Forward:	ACAAGGAACACCACGGAGAAC
	Reverse:	TCAAGCACGGAGGTACATCAC
Cxcl12	Forward:	CAAGATGTGAGAGGTGTGAGTC
	Reverse:	GCAGAGGAAGTGGCTATGGG
Il10	Forward:	GGAGTGAAGACCAGCAAAGG
IL1β (mice)	Forward:	TGTGCTCTGCTTGTGAGGTGCTG
	Reverse:	GCCGTGGTTGGAGAGATAGG
IL6 (mice)	Forward:	TAGTCCTTCCTACCCCAATTTCC
	Reverse:	TTGGTCCTTAGCCACTCCTTC
TNFα(mice)	Forward:	GGTGATCGGTCCCCAAAGGGATGA
	Reverse:	TGGTTTGCTACGACGTGGGCT
TGFβ(mice)	Forward:	TGGAGCCTGGACACACAGTA
	Reverse:	GTAGTAGACGATGGGCAGTGG
MMP9(mice)	Forward:	CACTGTCCACCCCTCAGAGC
	Reverse:	CACTTGTCGGCGATAAGG

Supplementary Figures

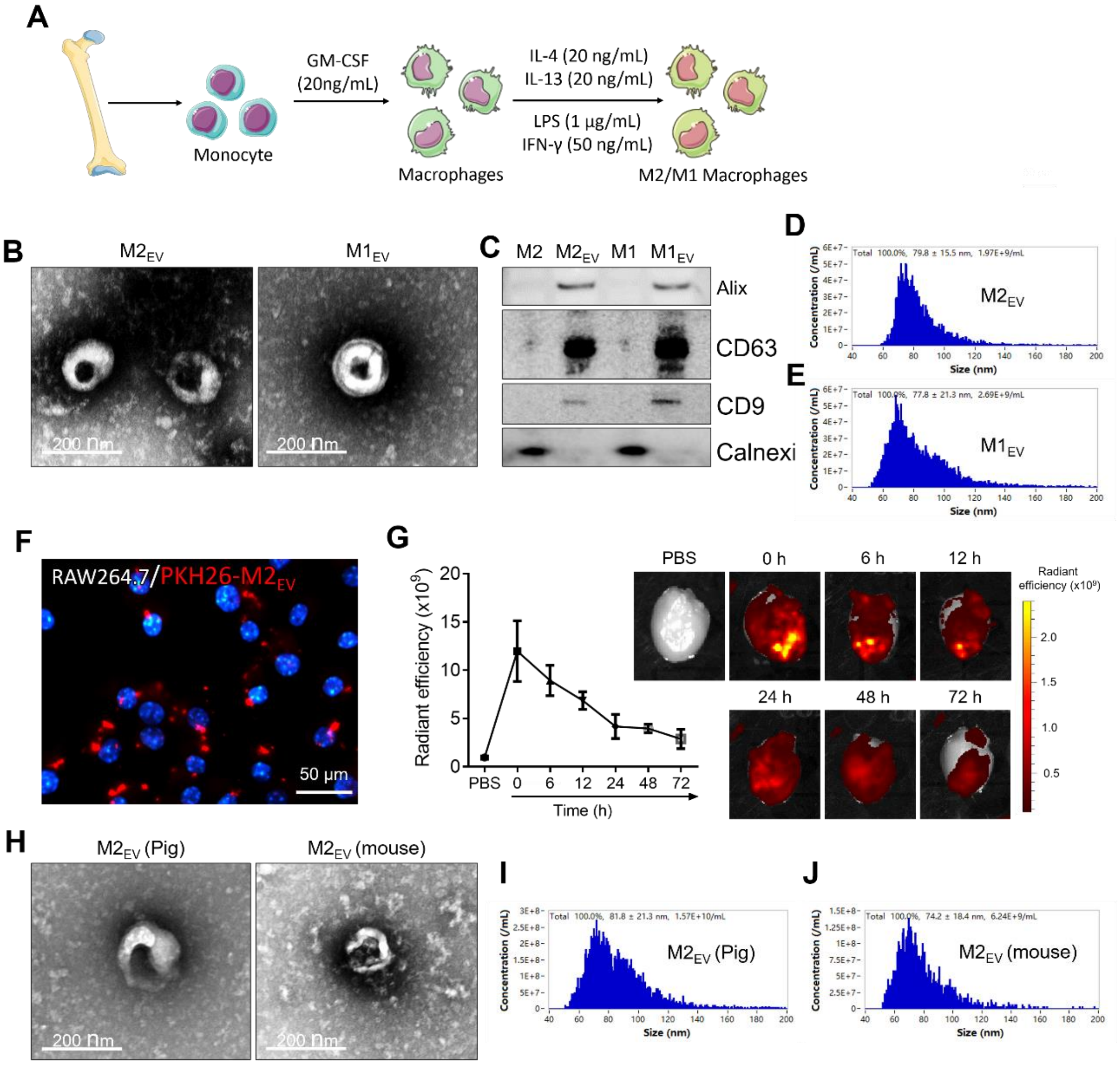

Figure S1. M2$_{EV}$ isolation, characterization, and bioavailability. (A) Schematic of the preparation of M1 and M2 BMDMs. **(B)** TEM visualization of dry-state rat M2$_{EV}$ and M1$_{EV}$. Scale bar = 200 nm. **(C)** Western blot detection to confirm presence of typical sEV/exosome markers and absence of endoplasmic reticulum marker, calnexin. **(D-E)** Nano-flow cytometry (NanoFCM) analysis of hydrodynamic diameter and concentration of rat M2$_{EV}$ and M1$_{EV}$. **(F)** M2$_{EV}$ uptake by RAW264.7 macrophages. Scale bars = 50 µm. **(G)** Near-infrared detection of intramyocardially injected PKH26-M2$_{EV}$ in the hearts of rats over the course of 72 h. **(H)** TEM visualization of dry-state pig M2$_{EV}$ and mouse M2$_{EV}$ **(I-J)** NanoFCM analysis of hydrodynamic diameter and concentration of pig M2$_{EV}$ and mouse M2$_{EV}$. All data are representative of n = 3 individual experiments.

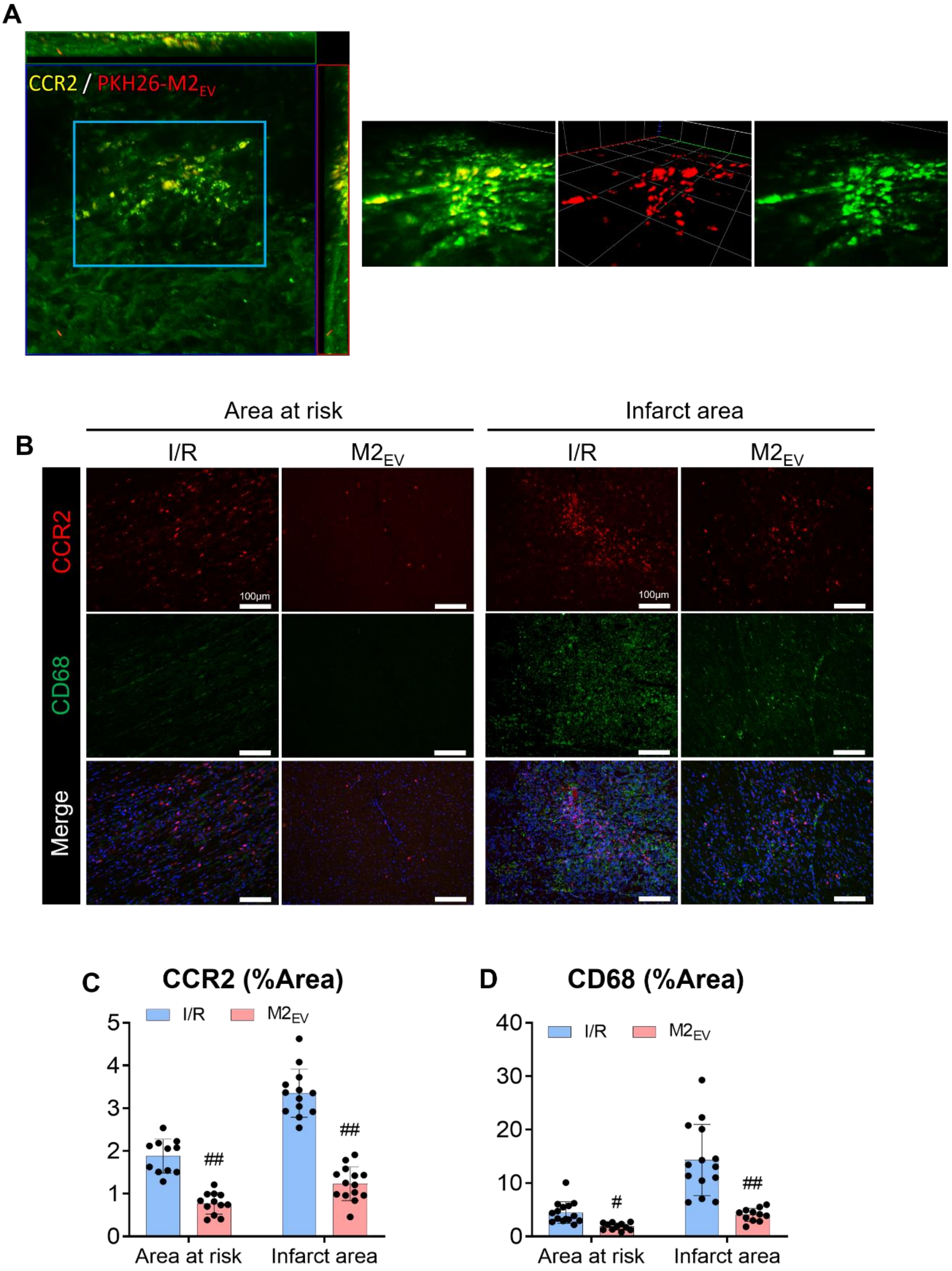

Fig. S2. M2$_{EV}$ inhibited CCR2^{+} macrophages in porcine I/R hearts. (A) Immunostaining of PKH26-M2$_{EV}$/CCR2 co-staining, respectively. **(B)** Immunostaining of CCR2 (red) and CD68 (green) in porcine myocardial tissue sections of the area at risk infarct border zone and the infarct area. Scale bar = 100 μm. **(C-D)** Quantification of CCR2 and CD68 immunostaining, 3 porcine models in each group, 3-5 microscopic fields of

view for each porcine sample, scale bar = 100 μm. Statistical significance is shown as $^{\#}p < 0.05$ and $^{\#\#}p < 0.01$ compared with the I/R group.

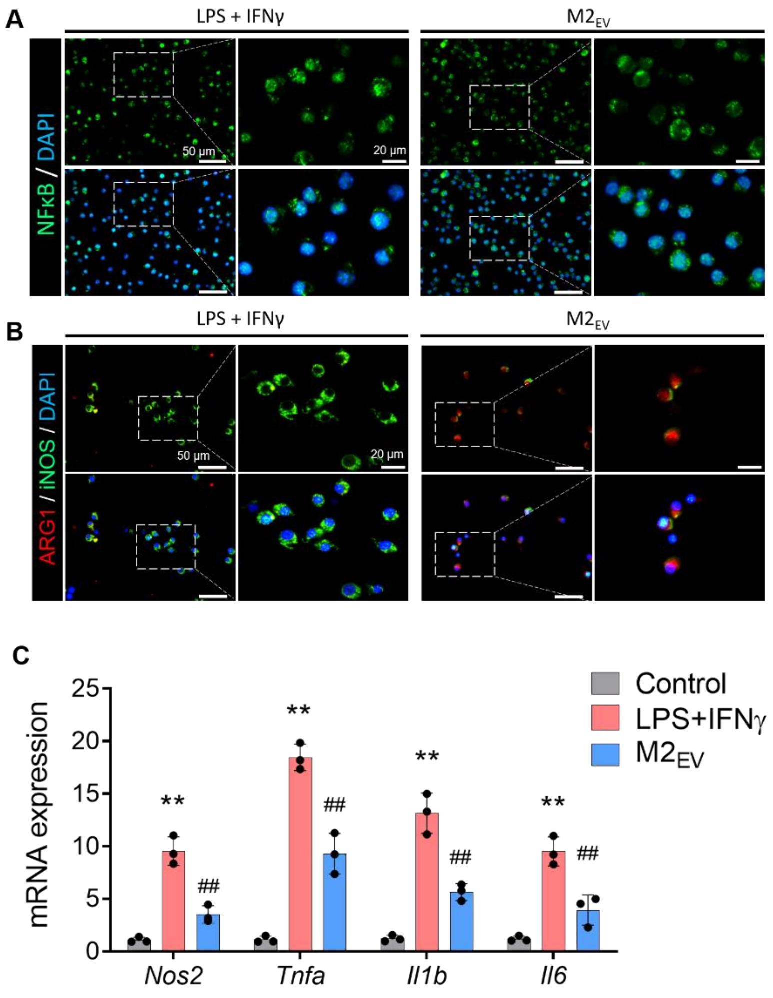

Fig. S3. $M2_{EV}$ treatment inhibits M1 macrophage activation and induces M2 macrophage polarization in vitro. (A) Representative immunocytochemistry images of NFκB expression and localization in treated BMDM. Scale bar = 50 μm. **(B)** Representative immunocytochemistry images of iNOS (green) and ARG-1 (red) in treated BMDM. Scale bar = 50 μm. **(C)** Relative mRNA expression of M1-associated genes, *Nos2*, *Il1b*, *Il6* and *Tnfa*. Data are presented as the mean ± SD of n = 3 individual experiments. Statistical significance is shown as $^{*}p < 0.05$ and $^{**}p < 0.01$ compared with the control group; $^{\#}p < 0.05$ and $^{\#\#}p < 0.01$ compared with the LPS+IFNγ group.

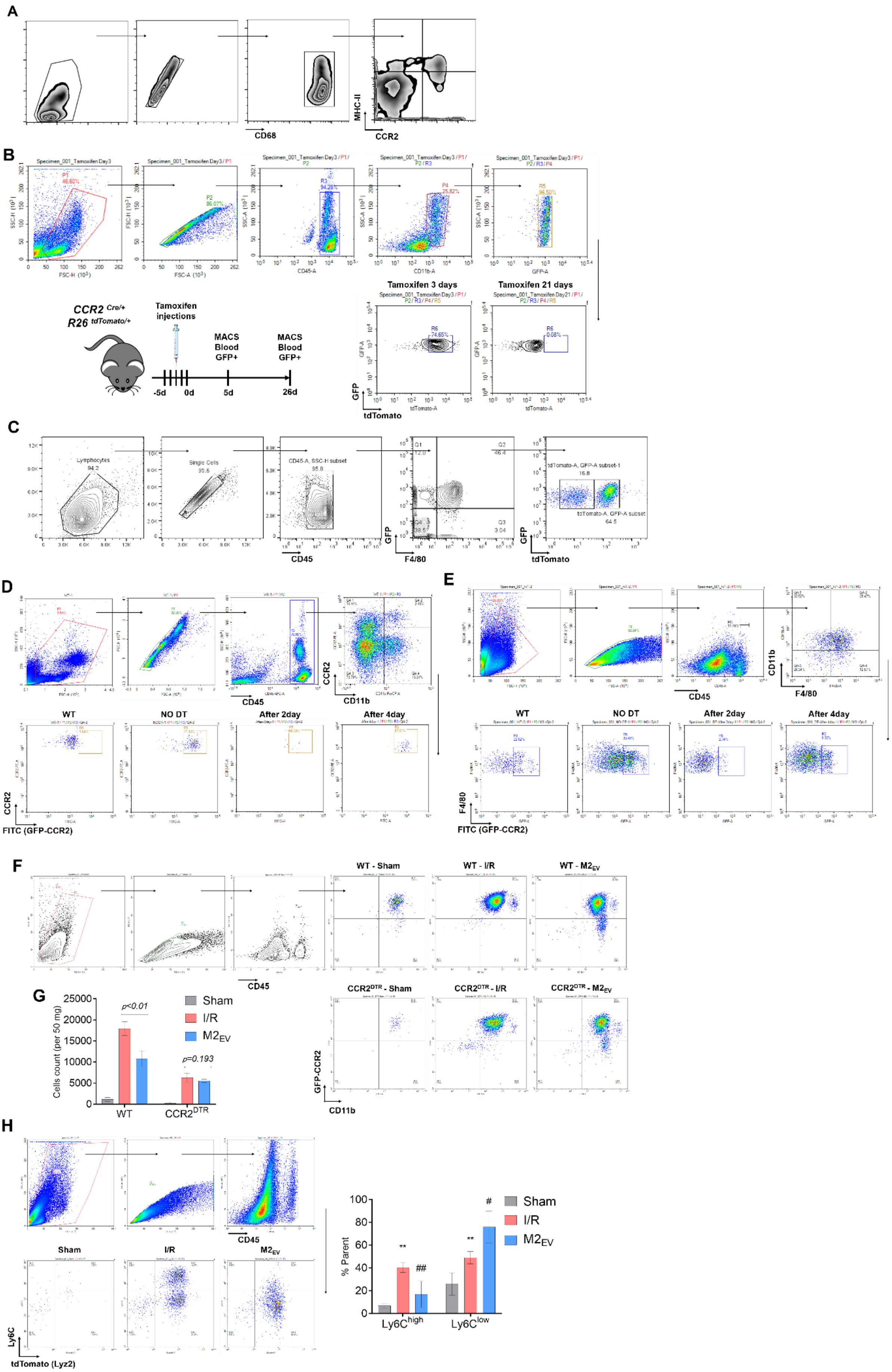
A
CD68
MHC-II
CCR2
B
Specimen_001_Tamoxifen Day3
P1
46.60%
P2
86.07%
R3
94.28%
P4
25.82%
R5
96.50%
FSC-H (10³)
FSC-A (10³)
SSC-H (10³)
SSC-A (10³)
CD45-A
CD11b-A
GFP-A
CCR2 Cre/+
R26 tdTomato/+
Tamoxifen injections
MACS
Blood
GFP+
-5d
0d
5d
26d
Tamoxifen 3 days
Tamoxifen 21 days
Specimen_001_Tamoxifen Day21 / P1 /
R6
74.65%
R6
0.08%
tdTomato-A
GFP
tdTomato
C
Lymphocytes
94.2
Single Cells
95.6
CD45-A, SSC-H subset
95.8
Q1
12.0
Q2
46.4
Q3
3.04
Q4
38.5
tdTomato-A, GFP-A subset-1
16.8
tdTomato-A, GFP-A subset
64.5
CD45
F4/80
GFP
tdTomato
D
CD45
CCR2
CD11b
WT
NO DT
After 2day
After 4day
CCR2
FITC (GFP-CCR2)
E
CD45
CD11b
F4/80
WT
NO DT
After 2day
After 4day
F4/80
FITC (GFP-CCR2)
F
CD45
WT - Sham
WT - I/R
WT - M2EV
CCR2DTR - Sham
CCR2DTR - I/R
CCR2DTR - M2EV
GFP-CCR2
CD11b
G
25000
20000
15000
10000
5000
0
Cells count (per 50 mg)
p<0.01
p=0.193
WT
CCR2DTR
Sham
I/R
M2EV
H
CD45
Sham
I/R
M2EV
Ly6C
tdTomato (Lyz2)
100
80
60
40
20
0
% Parent
**
##
**
#
Ly6Chigh
Ly6Clow
Sham
I/R
M2EV

Fig. S4. Schematic representation and results of flow cytometry workflow. (A) Flow cytometry assessment of rat MHC-II^{Hi}/CCR2^{+} macrophages. **(B)** Experimental design and flow cytometry assessment of tdTomato red fluorescence signal in CCR2^{+} monocytes isolated from blood after tamoxifen treatment. **(C)** Flow cytometry assessment of mouse resident and monocyte derived cardiac CCR2^{+} macrophages. **(D)** Assessment of GFP green fluorescence signal in CCR2^{+} monocytes from blood after DT treatment. **(E)** Assessment of GFP green fluorescence signal in CCR2^{+} monocytes from heart after DT treatment. **(F-G)** Quantification and assessment of CCR2^{+} monocytes from mouse heart after DT treatment, n = 3. **(H)** Quantification and assessment of myeloid derived monocytes, n = 4. Data are presented as the mean ± SD. Statistical significance is shown as $^{*}p < 0.05$ and $^{**}p < 0.01$ compared with the Sham group; $^{\#}p < 0.05$ and $^{\#\#}p < 0.01$ compared with the I/R group.

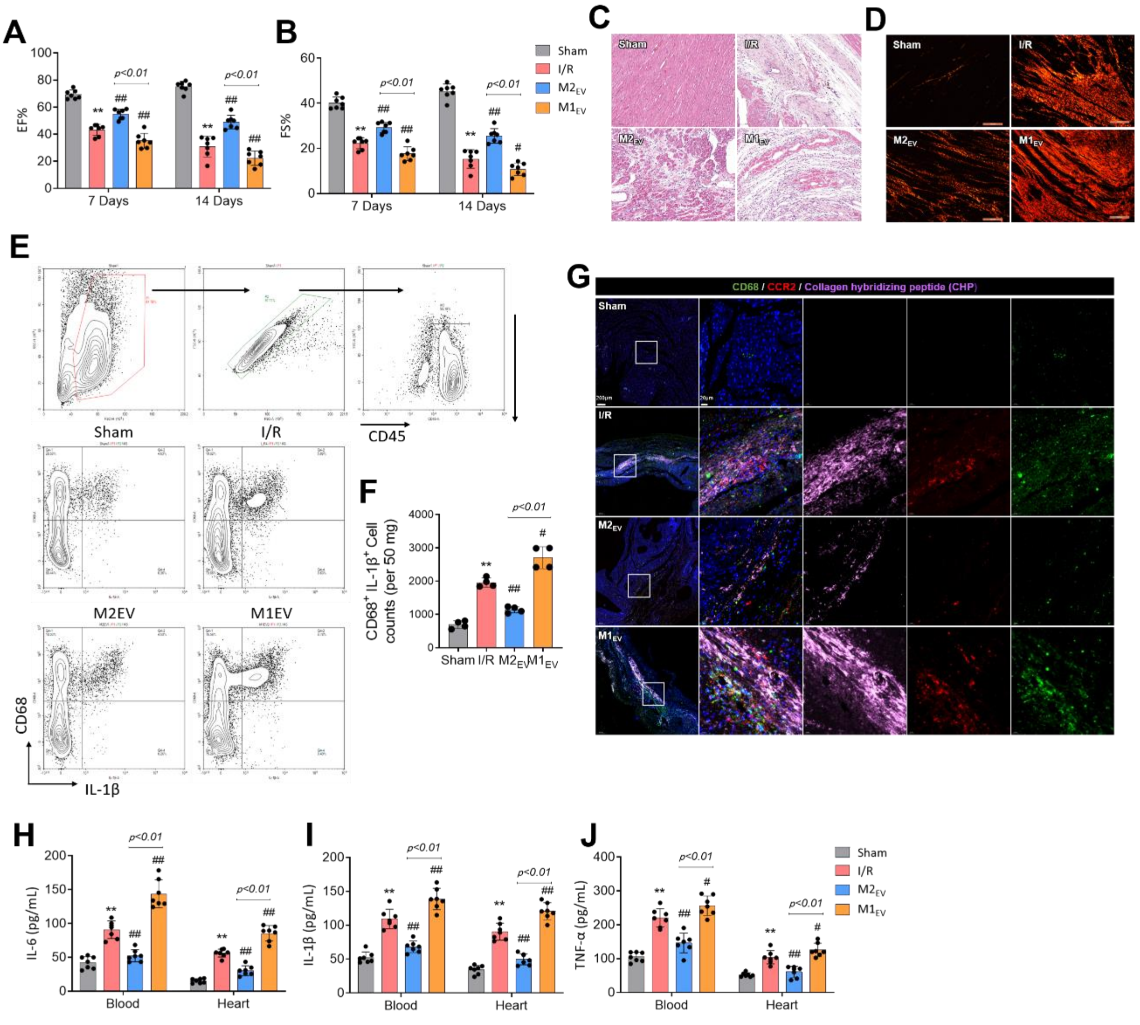

Fig. S5. Protective activity of M2$_{EV}$ in I/R rat hearts after 14days. (A) Quantification of **(B)** LVEF % and **(C)** LVFS %, n = 7. **(C)** Representative H&E staining of heart transverse sections (scale bar = 100 μm). **(D)** Representative Sirius Red staining of heart transverse sections (scale bar = 100 μm). **(E-F)** Quantification and assessment

of Proinflammatory macrophage from rat heart, n = 4. **(G)** Representative immunostaining of CD68, CCR2, and collagen hybridizing peptide. **(H-I)** The protein levels of IL-1β, IL-6 and TNF-α in rat heart using ELISA, n=7. All data are presented as the mean ± SD. Statistical significance is shown as: $^{**}p < 0.01$ compared with the sham group; and $^{\#\#}p < 0.01$ compared with the I/R group.

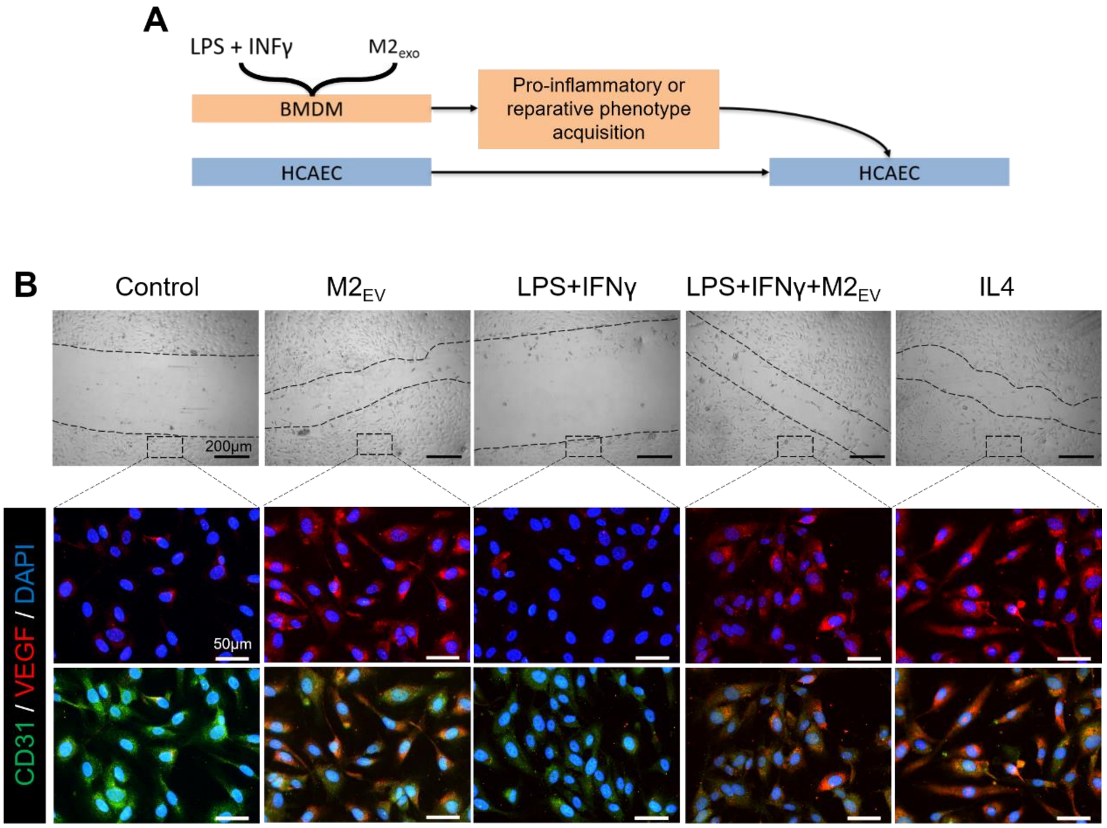

Fig. S6. M2$_{EV}$ promotes endothelial cell migration and VEGF expression. (A) Experimental procedure. **(B)** Scratch-wound assays of HCAEC in co-culture with differentially stimulated BMDM. Scale bar = 200 µm. **(C)** Representative immunocytochemistry images of CD31 and VEGF expression by HCAEC after co-culture with differentially stimulated BMDM. Scale bar = 50 µm. Images are representative of n = 3 individual experiments.

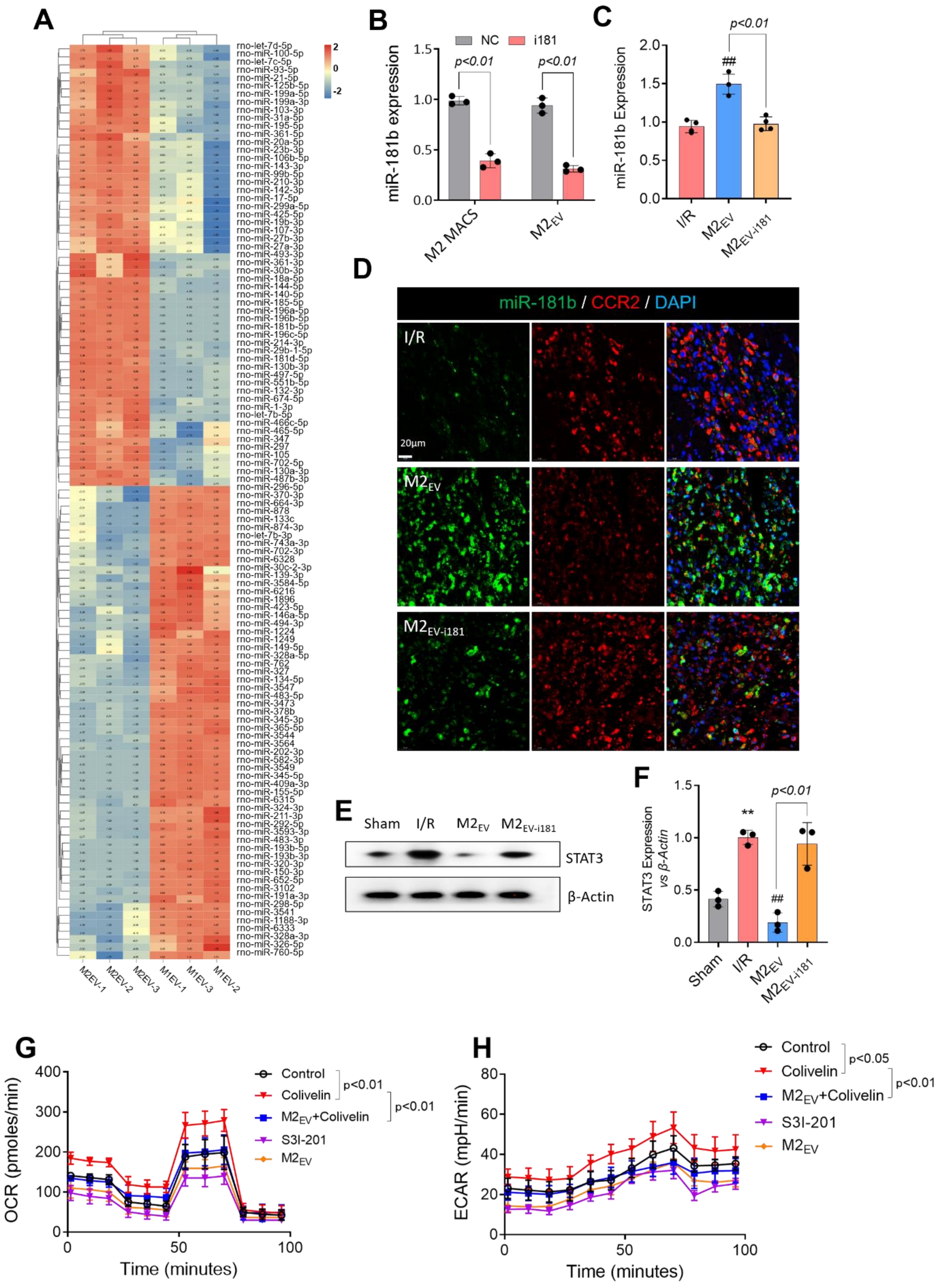

Fig. S7. MicroRNA-181b-5p has elevated expression in M2$_{EV}$. (A) Heat map and volcano plots displaying the differential expression of miRNAs in M2$_{EV}$ and M1$_{EV}$. **(B)** miR-181b-5p expression in M2 macrophages and M2$_{EV}$ with or without i181, n=3. **(C)**

miR-181b-5p expression in rat heart with or without i181, n-3. **(D)** Immunostaining of miR-181b/CCR2 co-staining (scale bar = 20 μm). **(E-F)** The protein level of STAT3 in $CCR2^+$ macrophages, as assessed using Western Blot and densitometric analysis, n=3. **(G-H)** OCR and ECAR measurements in $CCR2^+$ macrophages, as assessed by a Seahorse Bioscience XF24 analyzer, n=3. All data are presented as the mean ± SD. Statistical significance is shown as: $^{**}p < 0.01$ compared with the sham group; and $^{##}p < 0.01$ compared with the I/R group.

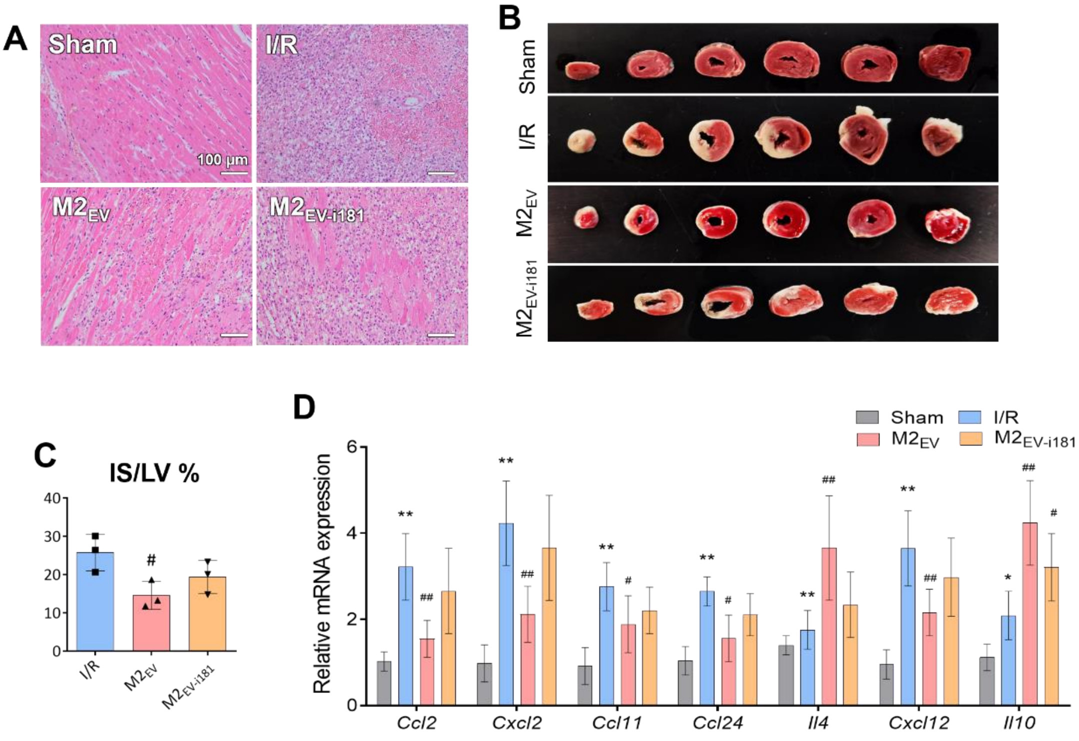

Fig. S8. MicroRNA-181b-5p is implicated in CCR2 regulation by $M2_{EV}$. (A) Representative H&E staining of transverse heart tissue sections. Scale bar = 100 μm. **(B-C)** Representative TTC stained heart sections and quantification of infarct site area relative to whole heart sections. **(D)** Relative mRNA expression of a panel of inflammation regulatory genes: *Ccl2*, *Cxcl2*, *Ccl11*, *Ccl24*, *Il4*, *Cxcl12*, and *Il10*. Data are presented as the mean ± SD of n = 3 individual experiments. Statistical significance is shown as $^{*}p < 0.05$ and $^{**}p < 0.01$ compared with the control group; $^{#}p < 0.05$ and $^{##}p < 0.01$ compared with the I/R group.

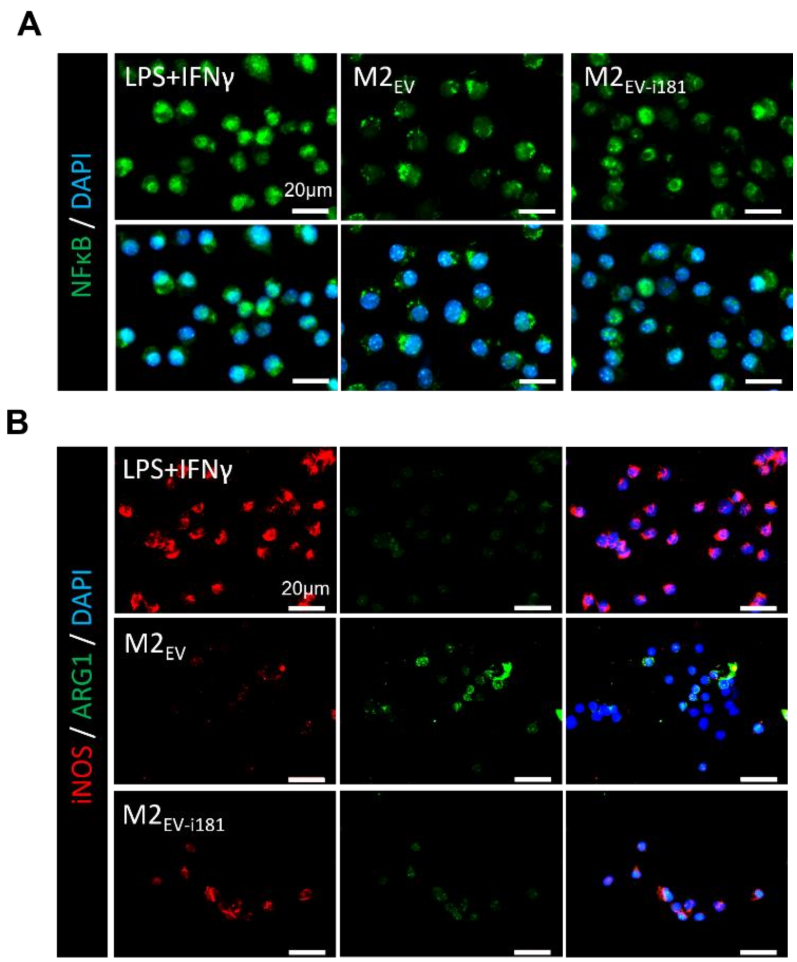

Fig. S9. MicroRNA-181b-5p involvement in macrophage phenotypic switching. (A) Representative immunostaining of NFκB localization in treated BMDM. Scale bar = 20 µm. **(B)** Representative immunostaining of iNOS (red) and ARG-1 (green) in treated BMDM. Scale bar = 20 µm. Images are representative of n = 3 individual experiments.

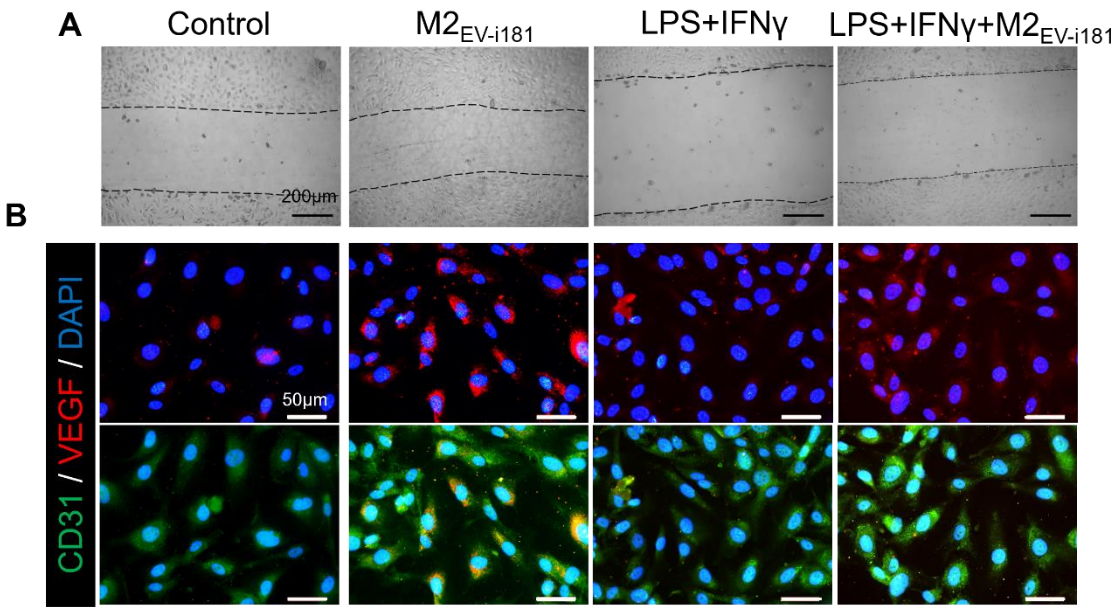

Fig. S10. MicroRNA-181b-5p involvement in macrophage-endothelial cell regulation. **(A)** Scratch wound migration assays of HCAEC co-cultured with treated BMDM. Scale bar = 200 µm. **(B)** Representative immunocytochemistry images of CD31 and VEGF expression in HCAEC co-cultured with treated BMDM. Scale bar = 50 µm. Images are representative of n = 3 individual experiments.

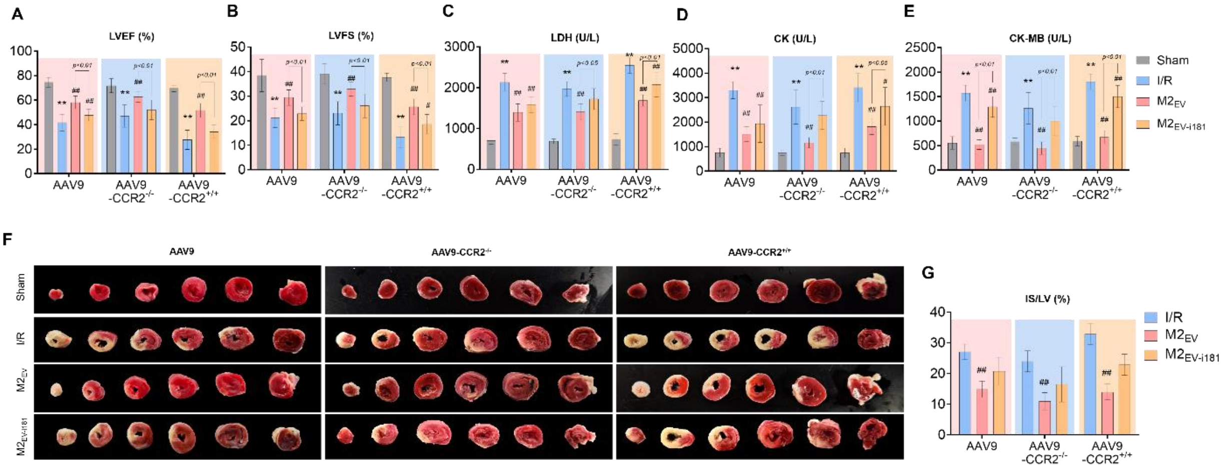

Fig. S11. Indiscriminate knockdown or overexpression of cardiac CCR2 does not influence M2$_{EV}$ therapeutic effects. (A-B) Quantification of LVEF and LVFS, n = 6. **(C-E)** Levels of LDH, CK, and CK-MB. **(F-G)** Representative TTC stained heart sections and quantification, n = 3. All data are presented as the mean ± SD. Statistical significance is shown as $^{*}p < 0.05$ and $^{**}p < 0.01$ compared with the control group; $^{\#}p < 0.05$ and $^{\#\#}p < 0.01$ compared with the I/R group.
